# Supplementary material for: MeNet: A mixed-effect deep neural network for multi-environment genomic prediction of agronomic traits
Source: Plant Commun. 2025 Nov 19;7(3):101620. doi: 10.1016/j.xplc.2025.101620 (PMC12983247; doi:10.1016/j.xplc.2025.101620)
Supplement: Document S1. Supplemental Figures 1–10 and Supplemental Tables 1–6 [file mmc1.pdf]

Plant Communications, Volume *7*

Supplemental information

MeNet: A mixed-effect deep neural network for multi-environment genomic prediction of agronomic traits

Yanhui Li, Shengjie Ren, Jixiang Li, Jiyong Lee, Jianmin Wan, and Xiangchao Gan

Supplementary information

Mixed-effect deep neural network for multi-environment genomic prediction of agronomic traits

Yanhui Li[1†], Shengjie Ren[1†], Jixiang Li[1], Jiyong Lee[1], Jianmin Wan[2,3*], Xiangchao Gan[1,2*]

[1]State Key Laboratory for Crop Genetics and Germplasm Enhancement and Utilization, Jiangsu Nanjing National Field Scientific Observation and Research Station for Rice Germplasm, Key Laboratory of Biology, Genetics and Breeding of Japonica Rice in Mid-lower Yangtze River, Ministry of Agriculture and Rural Affairs, Academy for Advanced Interdisciplinary Studies, Nanjing Agricultural University, Nanjing 210095, China.

[2]Zhongshan Biological Breeding Laboratory, Nanjing 210095, China.

[3]State Key Laboratory of Crop Gene Resources and Breeding, Institute of Crop Sciences, Chinese Academy of Agricultural Sciences, Beijing, China.

Supplementary Table 1| Prediction performance (R^2) of 11 models across 36 tests, including 29 rice traits evaluated under three environments, three maize traits, and wheat GY evaluated under four environments.

	Phenotype	RF	XGBoost	BayesC	rrBLUP	SoyDNGP	WheatGP	GPformer	Cropformer	CLCNet	VMGP	DeepCCR	MeNet
Rice for Shanghai	CL	0.802	0.828	0.805	0.803	0.803	0.802	0.795	0.803	0.804	0.812	0.814	0.833
	GL	0.645	0.713	0.711	0.726	0.676	0.669	0.684	0.688	0.692	0.721	0.721	0.726
	GW	0.630	0.686	0.677	0.686	0.621	0.629	0.630	0.599	0.593	0.693	0.688	0.701
	GY	0.352	0.333	0.352	0.352	0.282	0.321	0.286	0.334	0.351	0.352	0.348	0.357
	HD	0.697	0.706	0.645	0.651	0.658	0.657	0.674	0.667	0.678	0.709	0.703	0.725
	LA	0.173	0.174	0.169	0.172	0.171	0.167	0.175	0.182	0.177	0.176	0.178	0.182
	LL	0.327	0.347	0.353	0.353	0.303	0.297	0.297	0.341	0.348	0.337	0.308	0.358
	LW	0.365	0.435	0.442	0.443	0.394	0.399	0.379	0.425	0.388	0.443	0.420	0.457
	PL	0.386	0.414	0.414	0.411	0.384	0.375	0.399	0.393	0.397	0.404	0.402	0.421
	PN	0.267	0.282	0.299	0.297	0.274	0.287	0.275	0.291	0.298	0.279	0.263	0.300
	PH	0.795	0.809	0.792	0.793	0.786	0.785	0.789	0.792	0.792	0.796	0.809	0.817
	GPC	0.270	0.300	0.303	0.303	0.286	0.276	0.271	0.280	0.282	0.309	0.293	0.313
Rice for Hainan	CL	0.743	0.769	0.760	0.760	0.745	0.750	0.753	0.752	0.763	0.765	0.759	0.772
	HD	0.643	0.658	0.636	0.639	0.637	0.645	0.650	0.633	0.635	0.641	0.648	0.676
	LA	0.259	0.255	0.259	0.258	0.233	0.246	0.223	0.256	0.250	0.259	0.195	0.262
	LL	0.272	0.269	0.269	0.271	0.247	0.252	0.256	0.261	0.268	0.257	0.268	0.275
	LW	0.322	0.409	0.406	0.411	0.326	0.362	0.369	0.373	0.347	0.405	0.395	0.416
	PL	0.361	0.393	0.397	0.399	0.367	0.371	0.405	0.394	0.394	0.371	0.364	0.407
	PN	0.282	0.294	0.298	0.302	0.257	0.276	0.283	0.287	0.289	0.280	0.258	0.303
	PH	0.740	0.769	0.756	0.759	0.748	0.744	0.763	0.764	0.762	0.769	0.755	0.777
Rice for Hangzhou	CL	0.777	0.788	0.773	0.782	0.782	0.772	0.779	0.783	0.784	0.770	0.786	0.792
	GY	0.304	0.306	0.309	0.307	0.307	0.296	0.300	0.296	0.307	0.293	0.307	0.315

	HD	0.571	0.534	0.497	0.498	0.498	0.524	0.514	0.525	0.536	0.543	0.548	0.550
	LA	0.197	0.185	0.195	0.195	0.195	0.140	0.179	0.170	0.189	0.199	0.130	0.201
	LL	0.262	0.268	0.271	0.275	0.275	0.264	0.255	0.244	0.269	0.272	0.264	0.277
	LW	0.272	0.295	0.314	0.305	0.305	0.267	0.242	0.287	0.276	0.267	0.285	0.307
	PL	0.286	0.314	0.321	0.321	0.321	0.297	0.278	0.304	0.305	0.318	0.302	0.322
	PN	0.177	0.186	0.182	0.183	0.183	0.174	0.172	0.169	0.189	0.168	0.174	0.196
	PH	0.771	0.776	0.768	0.774	0.774	0.768	0.763	0.771	0.776	0.759	0.773	0.782
Maize	DTT	0.827	0.872	0.882	0.895	0.883	0.880	0.875	0.888	0.894	0.891	0.895	0.898
	EW	0.547	0.589	0.592	0.613	0.564	0.604	0.579	0.603	0.602	0.606	0.600	0.617
	PH	0.849	0.880	0.876	0.891	0.883	0.880	0.884	0.887	0.888	0.883	0.869	0.895
Wheat	GY1	0.280	0.281	0.168	0.251	0.200	0.276	0.249	0.216	0.274	0.277	0.273	0.282
	GY2	0.184	0.160	0.165	0.217	0.188	0.240	0.203	0.219	0.224	0.252	0.264	0.294
	GY3	0.113	0.125	0.061	0.126	0.072	0.134	0.113	0.126	0.140	0.105	0.132	0.146
	GY4	0.102	0.180	0.075	0.195	0.158	0.175	0.146	0.164	0.172	0.178	0.182	0.197

Supplementary Table 2| Comparative evaluation of prediction errors with three representative phenotypes for MeNet, rrBLUP, and DeepCCR.

Phenotype	Model	NMSE	MAPE	RRMSE
	MeNet	0.643	0.270	0.394
GY	rrBLUP	0.686	0.272	0.396
	DeepCCR	0.660	0.280	0.406
	MeNet	0.687	0.049	0.069
GPC	rrBLUP	0.697	0.050	0.070
	DeepCCR	0.698	0.050	0.070
	MeNet	0.275	0.040	0.060
HD	rrBLUP	0.349	0.046	0.067
	DeepCCR	0.290	0.047	0.061

Supplementary Table 3| Number of remaining SNPs under different LD filtering thresholds.

r^2 threshold	0.1	0.2	0.3	0.4	0.5
Retained SNP count	2280	5885	14385	25556	43660

Supplementary Table 4| The mean wall times (s) for trait prediction with seven deep neural networks.

Model	GY	HD	LA	Mean
MeNet	11.304	10.066	9.037	10.136
VMGP	7.155	6.988	5.973	6.705
DeepCCR	8.321	7.280	7.101	7.567
SoyDNGP	22.444	24.789	22.832	23.355
WheatGP	9.399	8.943	8.848	9.063
GPformer	23.755	23.717	23.316	23.596
Cropformer	7.346	7.188	6.244	6.926

Supplementary Table 5| Ablation experiments on 12 rice traits from the Shanghai validate the effectiveness of VE and RepGeno.

Phenotype	VE	RepGeno	Phenotype	VE	RepGeno
CL	0.828	0.805	LL	0.351	0.347
GL	0.703	0.661	LW	0.445	0.166
GW	0.693	0.612	PL	0.416	0.400
GY	0.345	0.328	PN	0.287	0.272
HD	0.719	0.659	PH	0.809	0.794
LA	0.181	0.137	GPC	0.306	0.229

Supplementary Table 6| Reference hyperparameter configurations for MeNet on the phenotypic data of 12 rice traits from Shanghai.

Phenotype	bath size	learning rate	channels of VE	embedding dim of VE	embedding dim of RepGeno	margin
CL	32	0.01	[1, 4, 16]	[1024, 1024]	[8192, 1024]	0.4
GL	32	0.01	[1, 4, 16]	[4096, 1024]	[4096, 1024]	0.1
GW	128	0.01	[1, 1, 1]	[8192, 1024]	[8192, 1024]	0.1
GY	128	0.01	[1, 1, 1]	[8192, 1024]	[8192, 1024]	0.1
HD	128	0.01	[8, 16, 64]	[1024, 1024]	[8192, 1024]	0.3
LA	128	0.01	[1, 1, 1]	[8192, 1024]	[8192, 1024]	0.1
LL	128	0.01	[2, 4, 8]	[1024, 1024]	[8192, 1024]	0.2
LW	128	0.01	[1, 1, 1]	[8192, 1024]	[8192, 1024]	0.1
PL	128	0.01	[2, 4, 8]	[1024, 1024]	[8192, 1024]	0.2
PN	128	0.01	[2, 4, 8]	[1024, 1024]	[8192, 1024]	0.1
PH	32	0.01	[1, 4, 16]	[1024, 1024]	[8192, 1024]	0.1
GPC	128	0.01	[1, 1, 1]	[8192, 1024]	[8192, 1024]	0.1

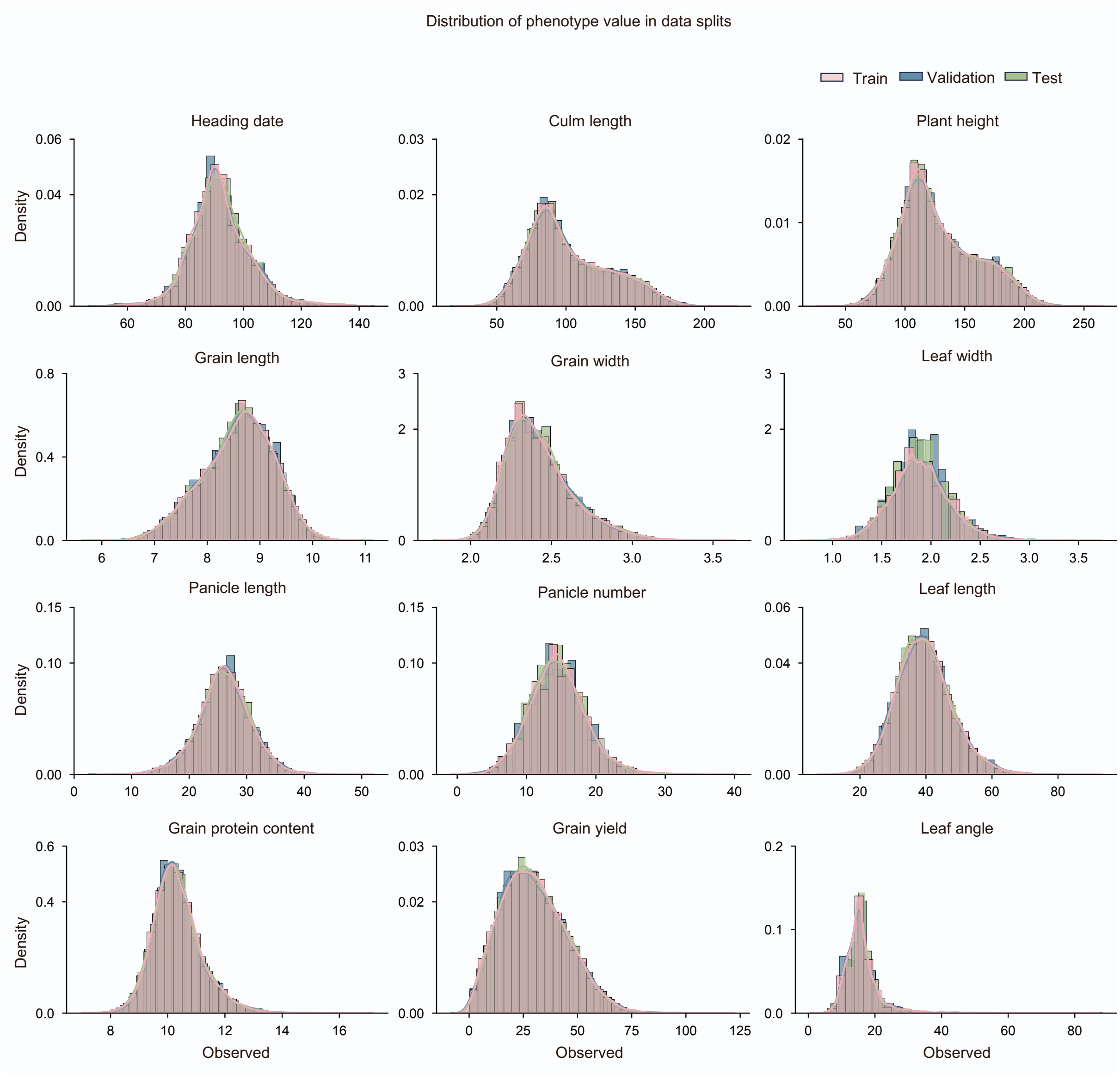

Supplementary Figure 1. Distribution comparison of each phenotype across training, validation, and test sets in the Shanghai dataset. Histograms represent frequency distribution, while KDE curves provide smoothed probability density estimates.

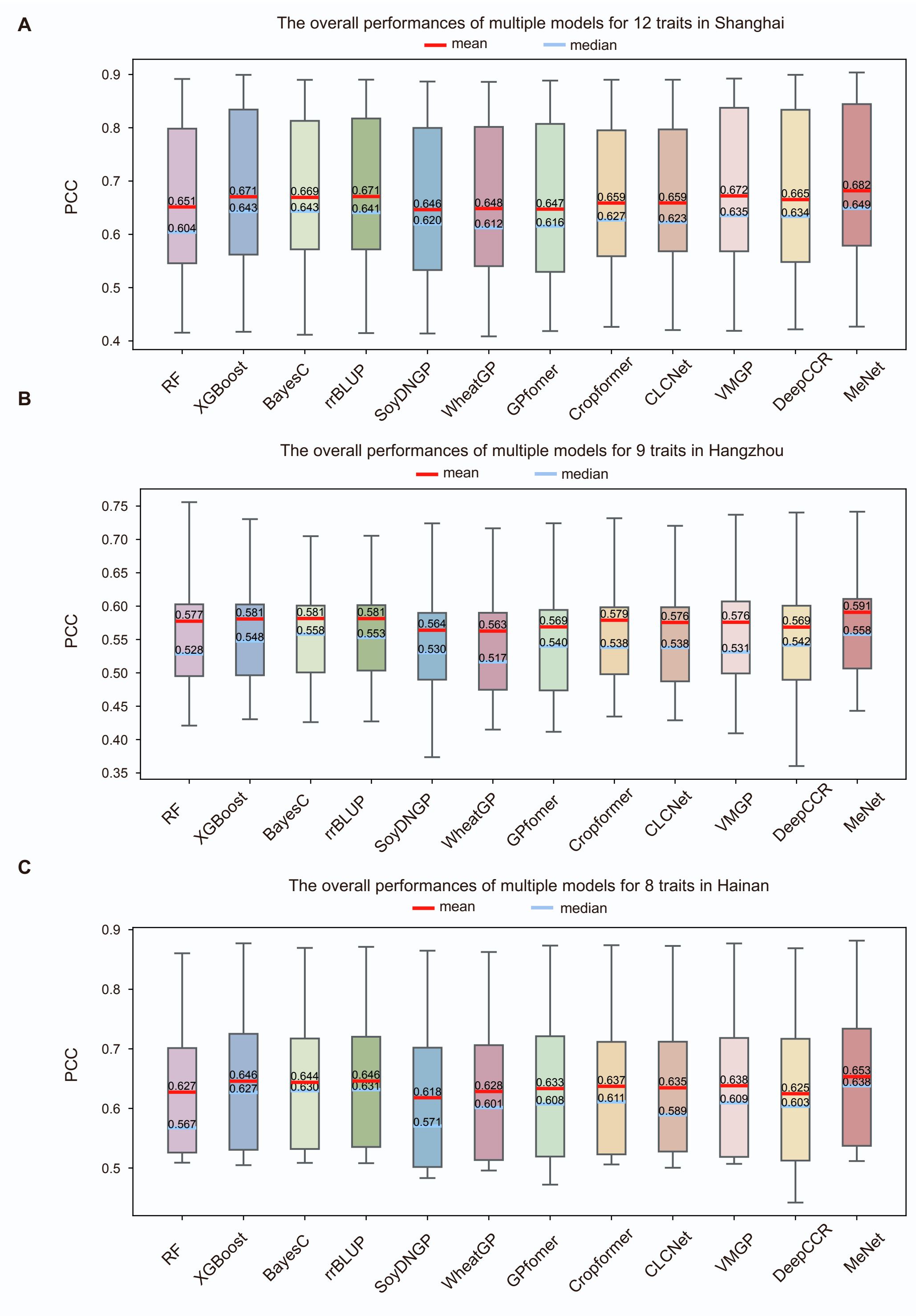

Supplementary Figure 2. Boxplots of the Pearson correlation coefficient (PCC) across multiple models for 12 traits in Shanghai (A), 9 traits in Hangzhou (B), and 8 traits in Hainan (C), using a 6:2:2 split for train set: validation set: test set. The red and blue lines indicate the mean and median PCC values, respectively.

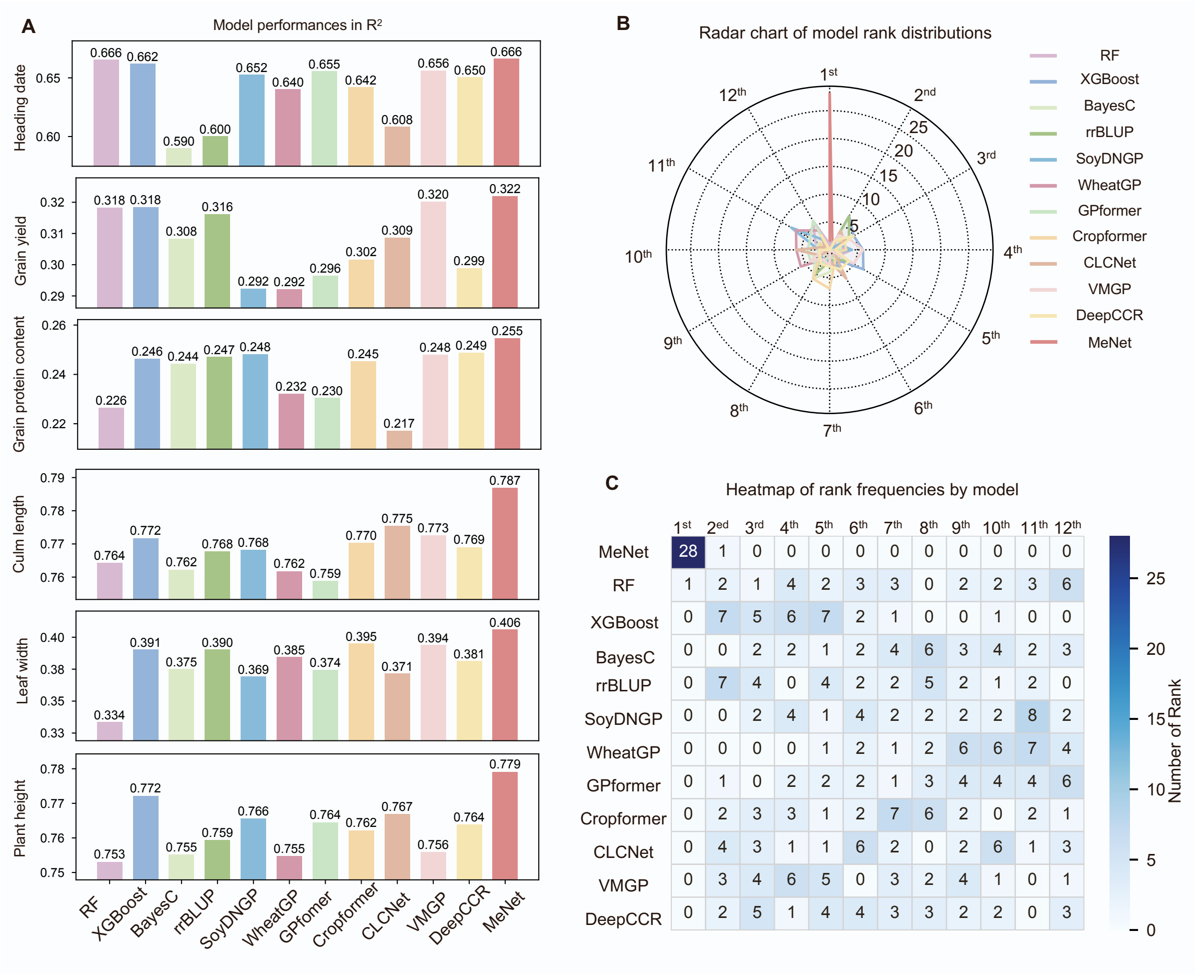

Supplementary Figure 3. Performances of MeNet for multiple rice traits under 3 environments with 3:2:5 for train set: validation set: test set. A, Bar charts showing the predictive ability (R^2) of MeNet compared with RF, XGBoost, BayesC, rrBLUP, SoyDNGP, WheatGP, GPformer, Cropformer, CLCNet, VMGP, and DeepCCR across rice traits in Shanghai. B-C, Statistical plots of ranks of predictive performances in R^2 for 29 tests by 11 models in the radar chart (B) and the heatmap (C).

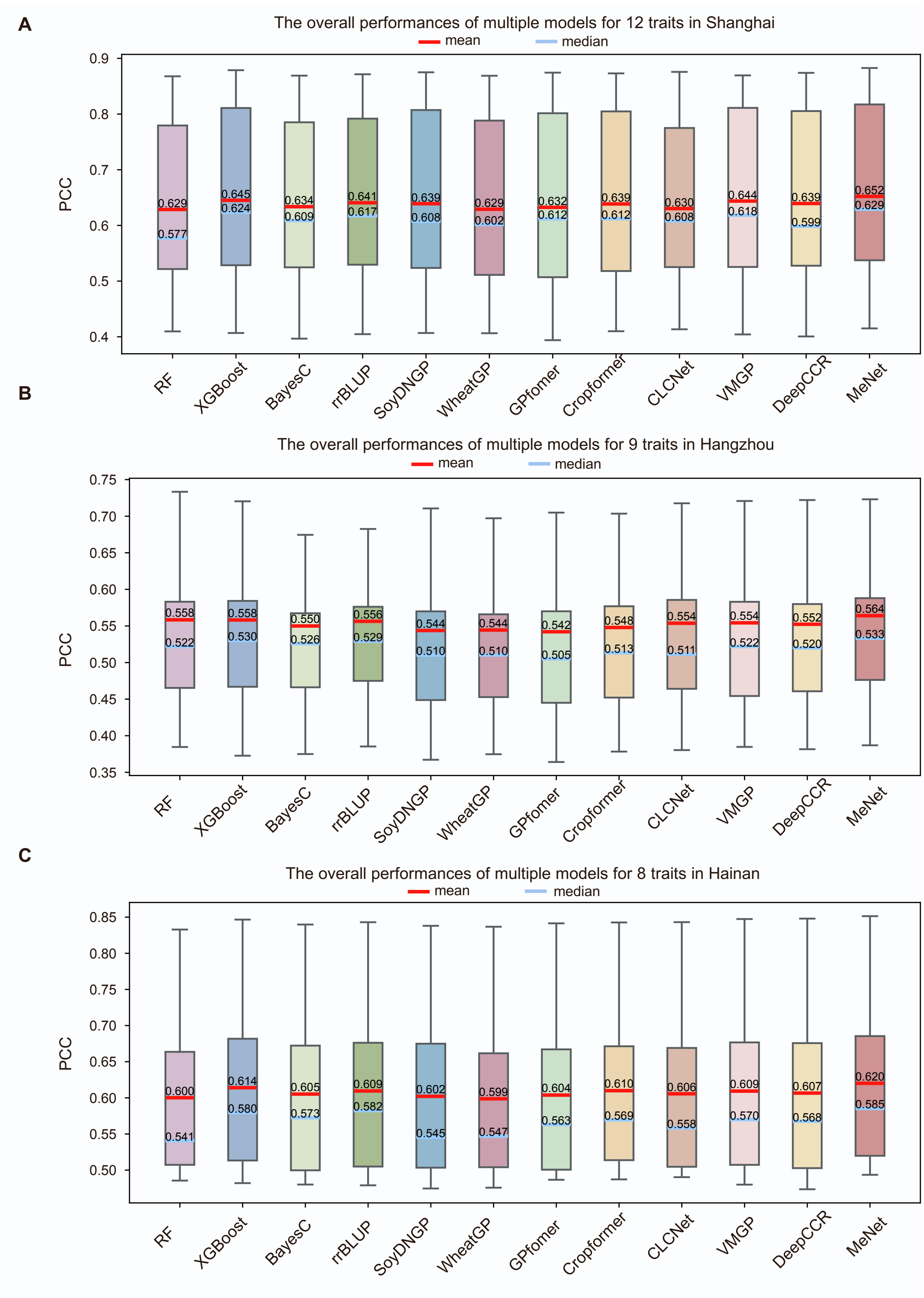

Supplementary Figure 4. Boxplots of the Pearson correlation coefficient (PCC) across multiple models for 12 traits in Shanghai (A), 9 traits in Hangzhou (B), and 8 traits in Hainan (C), using a 3:2:5 split for train set: validation set: test set. The red and blue lines indicate the mean and median PCC values, respectively.

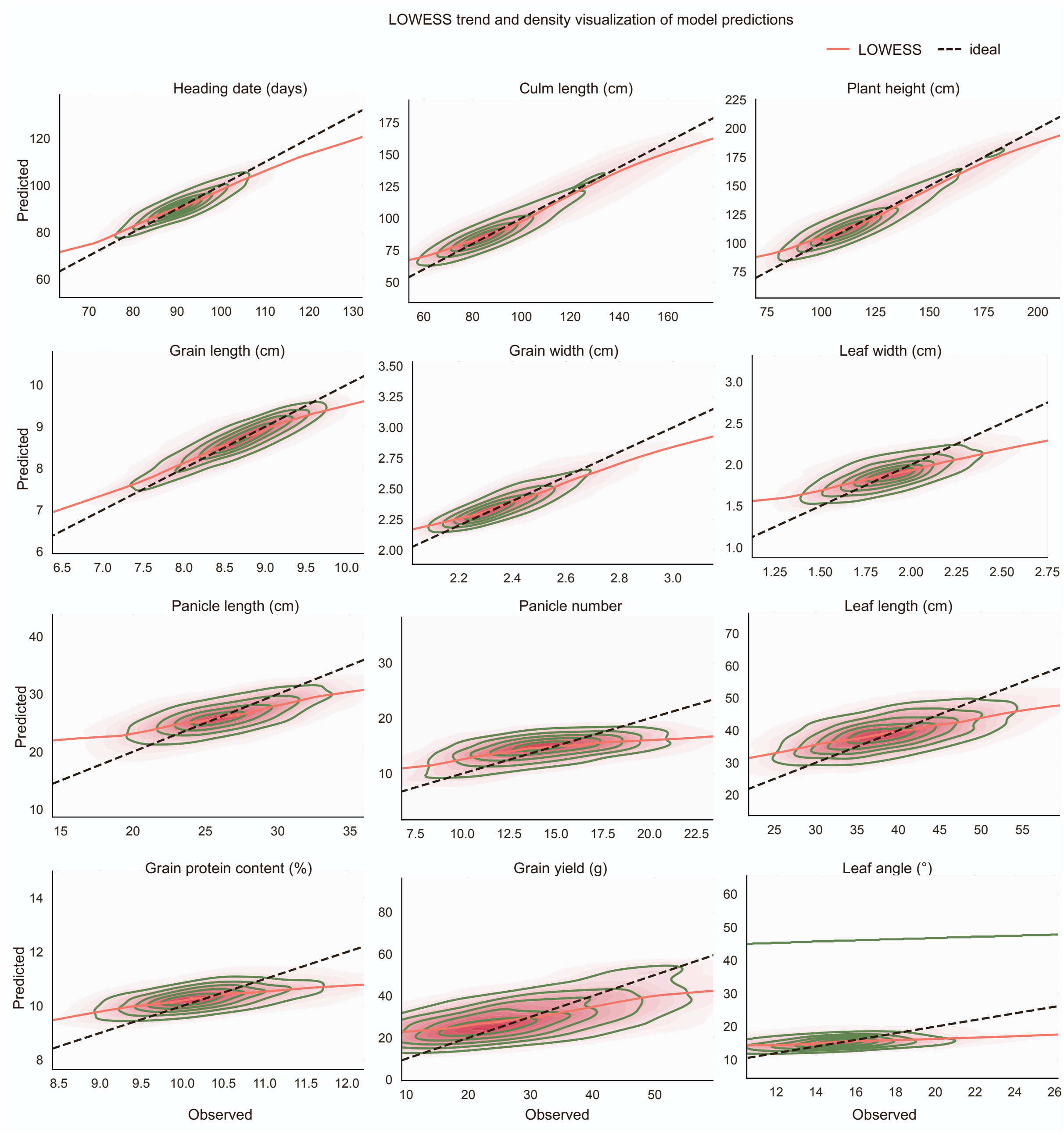

Supplementary Figure 5. The background is a 2D kernel density heatmap indicating data distribution in Shanghai dataset. The red curve represents the LOWESS fit, and the black dashed line indicates the ideal prediction ($y = x$).

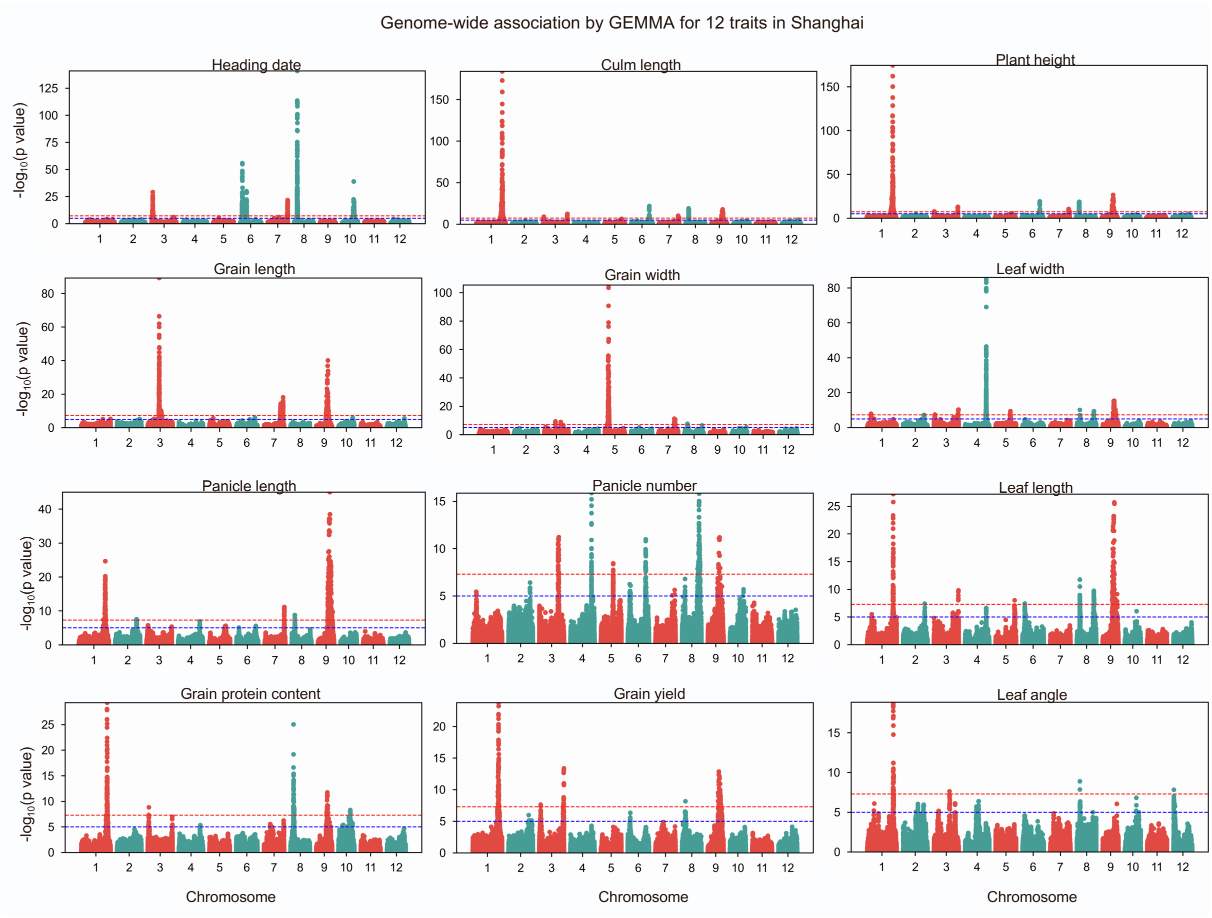

Supplementary Figure 6. Manhattan plots of genome-wide association analyses performed by GEMMA for 12 traits in Shanghai.

MeNet Software tools for Agronomic Trait Prediction

The MeNet software integrates model training, prediction, and transfer learning within a unified framework. It supports custom data import, hyperparameter configuration, and model saving/loading. With an intuitive graphical user interface, the software streamlines the end-to-end modeling and inference workflow, eliminating the need for programming skills. Tailored for crop breeding applications, it significantly enhances selection efficiency and operational simplicity.

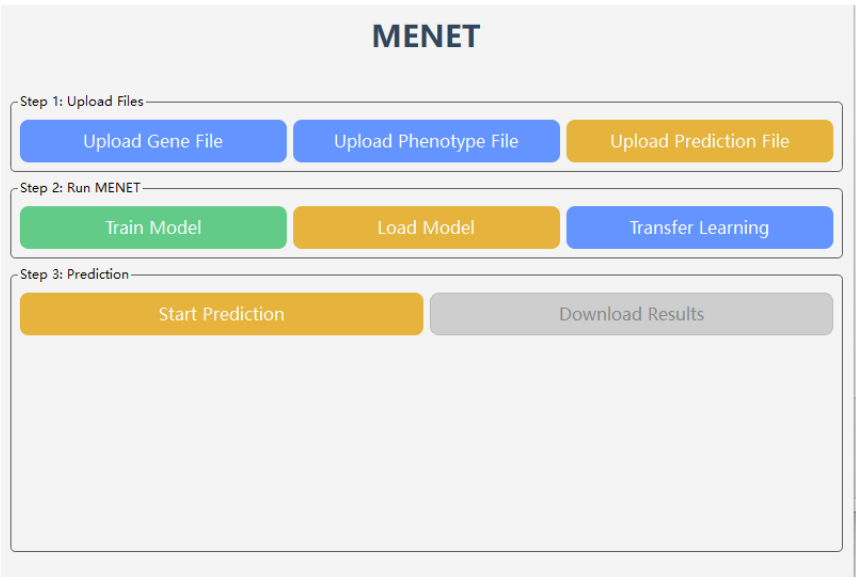

Supplementary Figure 7. The MeNet software user interface provides an intuitive and visually streamlined platform that facilitates seamless model training, dataset import, hyperparameter tuning, and inference.

Model training and saving

The software features a visual interface that enables users to flexibly configure training-related hyperparameters and import custom datasets. After training, the resulting models can be saved for subsequent loading, deployment, or transfer learning purposes.

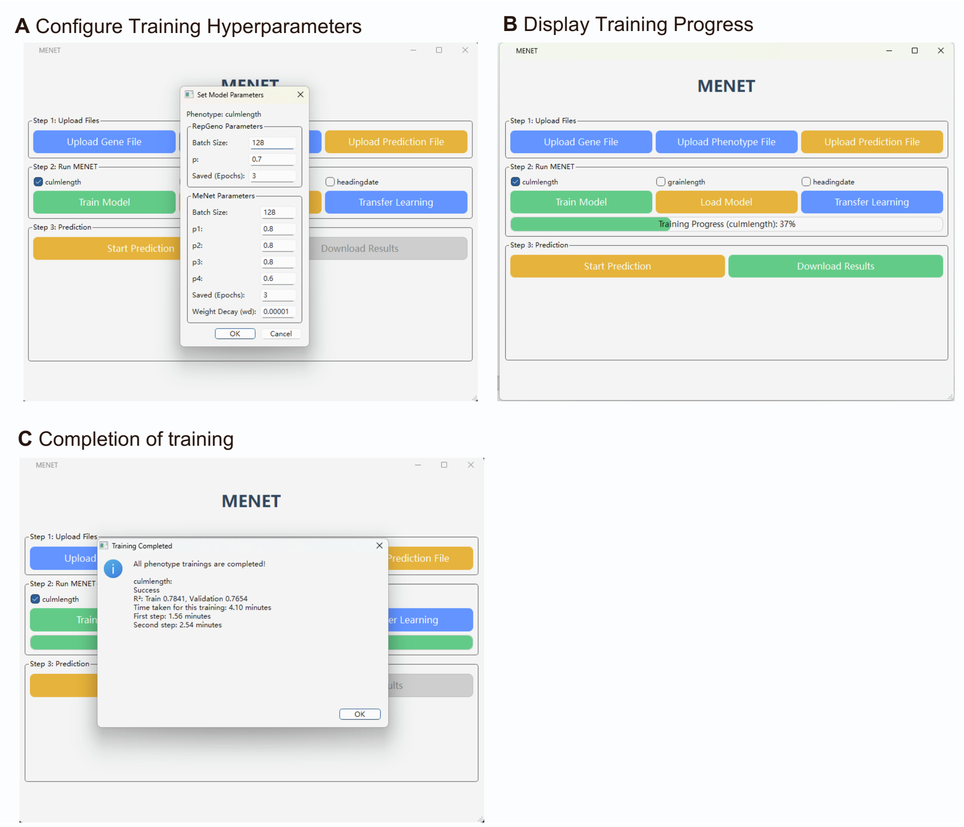

Supplementary Figure 8. (A) Hyperparameter configuration interface. (B) Visualization of training progress. (C) Notification indicating completion of training.

Model loading and inference

The software supports loading previously trained model files, enabling users to rapidly perform inference. Once a model is loaded, forward inference is automatically executed, and the prediction results are promptly displayed.

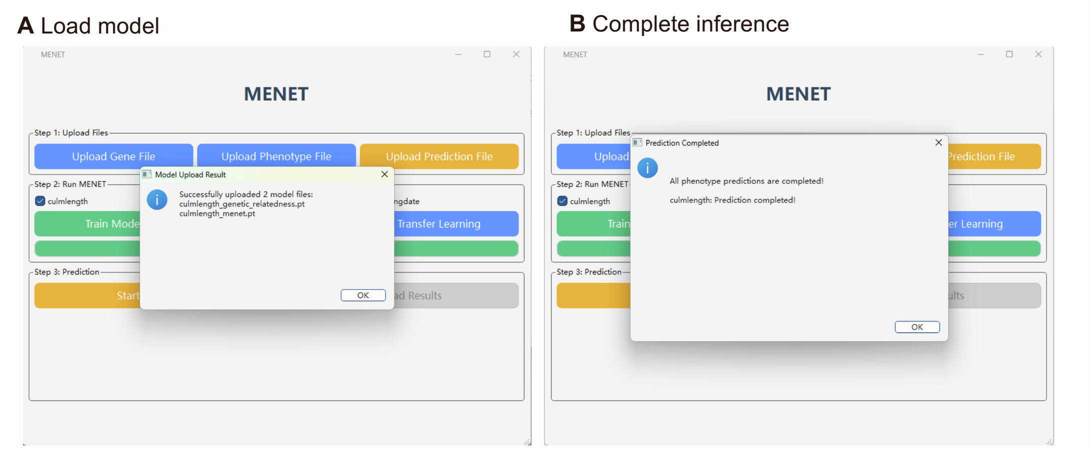

Supplementary Figure 9. (A) Interface for loading trained models. (B) Automatic inference.

Transfer Learning

The software enables transfer learning by allowing users to load previously trained models and fine-tune them on new datasets. Through a visual interface, users can configure parameters specific to the transfer learning process, which can be initiated with minimal effort. Upon completion, the updated model is automatically saved for future use and deployment.

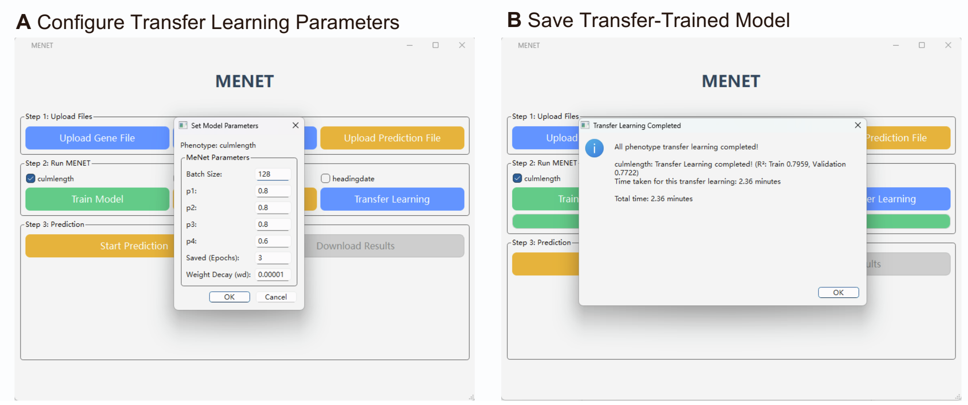

Supplementary Figure 10. (A) Interface for configuring transfer learning parameters. (B) Model saving after transfer learning.
