# Supplementary material for: MeNet: A mixed-effect deep neural network for multi-environment genomic prediction of agronomic traits
Source: Plant Commun. 2025 Nov 19;7(3):101620. doi: 10.1016/j.xplc.2025.101620 (PMC12983247; doi:10.1016/j.xplc.2025.101620)
Supplement: Document S2. Article plus supplemental information [file mmc2.pdf]

Plant Communications
Resource article

CellPress
Partner Journal

MeNet: A mixed-effect deep neural network for multi-environment genomic prediction of agronomic traits

Yanhui Li[1,4], Shengjie Ren[1,4], Jixiang Li[1], Jiyong Lee[1], Jianmin Wan[2,3,*] and Xiangchao Gan[1,2,*]

[1]State Key Laboratory for Crop Genetics and Germplasm Enhancement and Utilization, Jiangsu Nanjing National Field Scientific Observation and Research Station for Rice Germplasm, Key Laboratory of Biology, Genetics and Breeding of Japonica Rice in Mid-lower Yangtze River, Ministry of Agriculture and Rural Affairs, Academy for Advanced Interdisciplinary Studies, Nanjing Agricultural University, Nanjing 210095, China

[2]Zhongshan Biological Breeding Laboratory, Nanjing 210095, China

[3]State Key Laboratory of Crop Gene Resources and Breeding, Institute of Crop Sciences, Chinese Academy of Agricultural Sciences, Beijing, China

[4]These authors contributed equally to this article.

***Correspondence: Jianmin Wan (wanjianmin@caas.cn), Xiangchao Gan (gan@njau.edu.cn)**

https://doi.org/10.1016/j.xplc.2025.101620

ABSTRACT

Accurate genomic prediction of agronomic traits is critical for modern breeding and agriculture. Deep learning has great potential to enhance prediction by modeling nonlinearity, but its applications remain limited by inconsistent performance across diverse agronomic traits and environments and a lack of biological interpretability. Here, we propose a mixed-effect deep neural network (MeNet), a novel framework that unifies the statistical rigor of the mixed-effect model with the nonlinear modeling power of neural networks to advance genomic prediction. It employs dual embeddings to model phenotype-specific genetic relatedness as random effects and the cumulative nonlinear effect of genomic variants as fixed effects. Their contributions are dynamically adjusted through adaptive learning of the genetic complexity of the trait. When tested on three datasets from three crops, including 12 rice traits under three environments, wheat grain yield under four environments, and three maize traits, MeNet achieved superior performance in 34 of 36 evaluations, outperforming 11 state-of-the-art models, including conventional statistical models and deep learning approaches. Notably, it exceeded the theoretical upper bound of additive heritability for key traits, demonstrating its capacity to capture epistatic and gene-by-environment interactions. MeNet enables direct exploitation of genomic variants without dimensionality reduction and scales robustly with marker quantity. It performs cross-environment prediction using only 10% of field samples for training, achieving, on average, a 57.07% gain over baseline models. MeNet's robustness and generalizability underscore the potential for foundation models to streamline multi-site and multi-year prediction with minimal field data for the breeding of climate-resilient crops.

Key words: deep learning, genomic selection, trait prediction, transfer learning, nonlinear effects

Li Y., Ren S., Li J., Lee J., Wan J., and Gan X. (2026). MeNet: A mixed-effect deep neural network for multi-environment genomic prediction of agronomic traits. Plant Comm. **7**, 101620.

INTRODUCTION

Prediction of agronomic traits has wide applications in modern crop and animal breeding. Technological advances in high-throughput genotyping have enabled highly efficient prediction of phenotypic traits for large populations using genome-wide variants and phenotypic data from a subset of samples. The use of genomic prediction models to facilitate marker-assisted selection, trait introgression, and even genome editing in both crops and livestock has progressively come to perform a central and catalytic role in modern crop and animal breeding (Hayes and Goddard, 2010).

A mainstay in genomic prediction has been best linear unbiased prediction (BLUP) (Meuwissen et al., 2001), which efficiently exploits pedigree or genetic relatedness among samples. BLUP and its derivatives, including genomic BLUP (gBLUP), ridge regression BLUP (rrBLUP), compressed BLUP (cBLUP), and BayesBLUP, were introduced decades ago and remain among the most widely used genomic prediction tools in modern

 © 2025 The Authors. Published by Elsevier Inc. on behalf of CAS Center for Excellence in Molecular Plant Sciences, Chinese Academy of Sciences, Chinese Society for Plant Biology.
This is an open access article under the CC BY license (http://creativecommons.org/licenses/by/4.0/).

breeding (Meher et al., 2022). These models, especially rrBLUP, are robust and can provide reliable performance in predicting a variety of agronomic traits (Xiang et al., 2024). Other methods, including Lasso regression (Tibshirani, 1996) and Bayesian approaches (BayesA, BayesB, and BayesC) (De Los Campos et al., 2009; Gianola et al., 2009; Gianola, 2013), do not explicitly incorporate genetic relatedness information but instead extend the linear model to capture the functional impact of genomic variation through various prior distributions and regularization techniques. In general, the statistical models underlying these traditional methods are linear. Although they excel at modeling additive genetic effects, they inherently overlook higher-order interactions, such as gene-by-gene or gene-by-environment interactions.

In breeding, dominance effects and epistatic effects from gene–gene and gene–environment interactions make significant contributions to phenotypic divergence (Cheverud and Routman, 1995; Mackay, 2001; Rogers et al., 2021). Although these effects can theoretically be analyzed using linear models with specialized variant-coding schemes or interaction variables (Cheverud et al., 1999), it is often impractical to do so in genome-wide association or genomic prediction studies. These interactions typically exhibit high nonlinearity in phenotypic traits, and traditional statistical models often fail to adequately account for such effects, thereby markedly reducing the accuracy of trait prediction. To embrace nonlinearity for genomic prediction, machine learning (ML)-based algorithms have been proposed. Notable algorithms include support vector machines (Ogutu et al., 2011), decision trees, and random forests (RFs) (Montesinos López et al., 2022). Subsequent advances in learning strategies, such as those used by extreme gradient boosting (XGBoost) (Abdollahi-Arpanahi et al., 2020) and light gradient boosting machine (Yan et al., 2021), have significantly improved predictive performance.

In recent years, deep neural networks (DNNs) (Wang et al., 2023), such as convolutional neural networks (Ma et al., 2024), long short-term memory networks (Okut, 2021), and Transformer-based methods (e.g., DNABERT-2 and Nucleotide Transformer [Ji et al., 2021; Dalla-Torre et al., 2025; Zhou et al., 2024a]), have shown considerable potential for prediction tasks. These methods are particularly favored for modeling the functional impact of genomic variation and capturing nonlinear interactions and latent patterns within genotypic data (Wang et al., 2022). Most deep learning (DL) genomic prediction approaches focus on exploiting multi-scale information from genomic variants and depend heavily on dimensionality reduction to avoid overfitting (Du et al., 2018). Little attention is paid to genetic background information. Although DL methods have demonstrated significant promise in genomic prediction, they frequently suffer from inconsistent performance across different agronomic traits, and most importantly, they often lack biological interpretability (Zou et al., 2019). A comprehensive framework that exploits both linear and nonlinear relationships to account for the complexity of genetic architectures and integrates both genetic background information and the cumulative impact of genomic variation for prediction would greatly enhance our understanding of complex traits and genomic prediction.

Here, we propose a mixed-effect DNN (MeNet). It models genetic information with dual embeddings: one representing the genetic background as random effects and the other capturing the cumulative impact of genomic variants with nonlinear interactions as fixed effects. MeNet adaptively changes its weights during training through adaptive learning of the genetic complexity of the trait. Unlike most DL genomic prediction approaches that depend heavily on dimensionality reduction to avoid overfitting, the architecture of the mixed-effect model of MeNet enables straightforward exploitation of all genomic variants, thus offering high scalability. We applied MeNet to 36 predictions for three crops from three publicly available datasets. We then evaluated MeNet's robustness under data-limited scenarios and produced cross-environment predictions using 10% of the samples for training and validation, simulating the resource constraints faced in practical breeding programs.

RESULTS

An architecture designed for MeNet

The linear mixed-effects model (LMM) has wide applications in statistical analyses, including genome-wide association studies (GWASs) (the Million Veteran Program et al., 2018). However, traditional LMMs focus on additive genetic effects and cannot efficiently account for epistatic effects. The nonlinear mixed-effects model, which also allows for epistasis, can be formulated as

$$y = \beta_0 + \sum_i \beta_i X_i + \sum_{(i,j) \in C} \theta_{ij} X_i * X_j + Z\gamma + \epsilon,$$

where y is the phenotype, X_i is the *ith* genomic variant, i,j is a combination of interacting genomic variants, $Z\gamma$ is the random effect related to genetic relatedness, and ε *is* the noise or environmental factors. Unfortunately, the explicit incorporation of epistasis into LMMs in this manner is impractical, as the computational demand for exhaustive searching of pairwise interactions is extremely high, even for a moderate number of genetic markers.

Here, we model traits using a specially designed DNN, MeNet, to effectively capture the nonlinear cumulative effects of genomic variants (Figure 1A). In practice, most DNNs that have been applied to genomic prediction show promising performance in capturing nonlinearity. MeNet addresses two key limitations inherent to these networks. First, DNNs have an inherent demand for a large number of samples (Hollmann et al., 2025) and struggle to deliver stable results with the medium-sized datasets (often <10 000 training samples) or small datasets (often <2000) regularly used in breeding. The second limitation stems from the fact that dense markers are increasingly used for genomic prediction, as genome sequencing becomes a regular practice for genotyping. The enriched association among genomic markers, called linkage disequilibrium (LD), frequently leads to an increased computational load. As a consequence, existing DNNs depend heavily on dimensionality reduction.

MeNet employs a dual-branch architecture (Figure 1A), with one branch modeling fixed effects and the other modeling random effects, which represents genetic relatedness information among samples (referred to as RepGeno). RepGeno generates embeddings by capturing phenotype-specific genetic

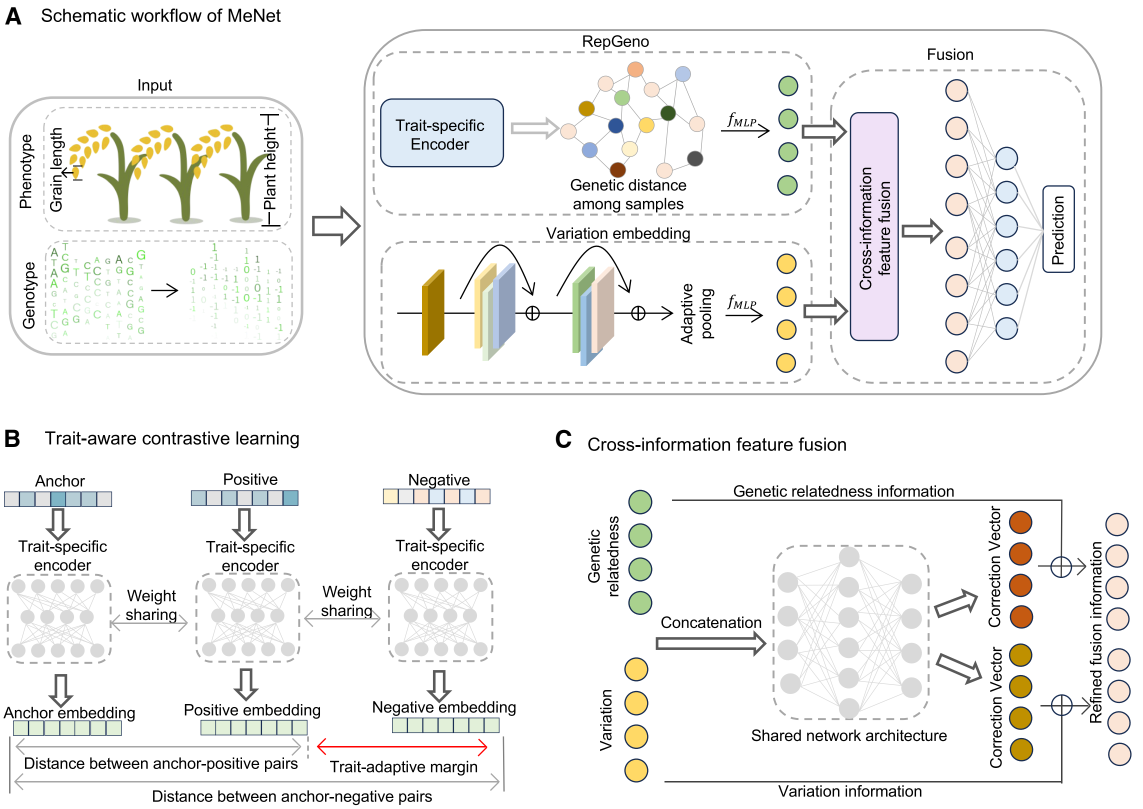

Figure 1. Overview of MeNet for trait prediction.

(A) The architecture of MeNet. The input consists of genomic variation data from the population and phenotypic data from a subset of samples. RepGeno is designed to extract embeddings representing genetic background information. In parallel, a CNN-based VE network extracts embeddings representing the cumulative impact of genomic variation. The embeddings from both modules are integrated for training with the observed phenotypic data, enabling accurate trait prediction.

(B) Trait-aware contrastive learning for training the RepGeno module.

(C) Details of the cross-information feature fusion unit of the fusion module.

relatedness via trait-aware triplet contrastive learning (Figure 1B), distinguishing it from the genetic relatedness matrix in LMMs by incorporating phenotypic information. In parallel, the variation embedding (VE) module, implemented as a residual-enhanced convolutional network, models the nonlinear effects of genomic variants. These two embeddings are integrated through the fusion module (Figure 1C), and MeNet dynamically adjusts their contributions with adaptive learning of the genetic complexity of the target phenotype. In practice, this novel architecture not only increases the stability of predictive performance for traits across multiple environments but also enhances robustness with medium sample sizes. The integration enhances the model's capacity to leverage both latent population genetic relatedness and variant signals, thereby supporting accurate modeling of complex genotype–phenotype associations. In both modules, the genomic markers are used in a straightforward manner. If the number of genomic markers is too large (>100 000 by default), chromosome-by-chromosome processing or a sliding window is used.

Performance of MeNet for multiple agronomic traits

We evaluated the overall performance of MeNet using three publicly available datasets. The first dataset is an 18 000-panel rice population (n = 18 421 accessions) that contains 15 recombinant inbred line populations and a four-way multiparent advanced generation inter-cross population (Wei et al., 2024). Numeric phenotypes for 12 agronomic traits were collected across three sites with different photoperiods and temperatures: Shanghai, Hangzhou, and Hainan. The other two datasets are a wheat dataset of grain yield (GY) (n = 599) across 4 environments (Crossa et al., 2010) and a maize dataset with 3 traits (n = *8652*) (Xiao et al., 2021). The detailed analyses are based on the rice dataset, which is the most comprehensive of the three. The rice traits include culm length (CL), plant height (PH), heading date (HD), GY, grain length (GL), grain width (GW), grain protein content (GPC), leaf length (LL), leaf width (LW), leaf angle (LA), panicle length (PL), and panicle number (PN). These traits are generally believed to differ significantly in genetic complexity. For instance, previous studies have shown that rice HD has very high heritability, and HDs of a population growing in the same field can be predicted accurately using only 11 genes (Zhang et al., 2019; Kawakita et al., 2024). By contrast, GY is one of the most challenging traits to predict, as it is a complex trait with low heritability that involves many small-effect genes, and it is heavily influenced by environmental and cultivation factors (Spindel et al., 2015).

We used the same datasets to compare MeNet with 11 representative models: RF, XGBoost, BayesC, rrBLUP, SoyDNGP

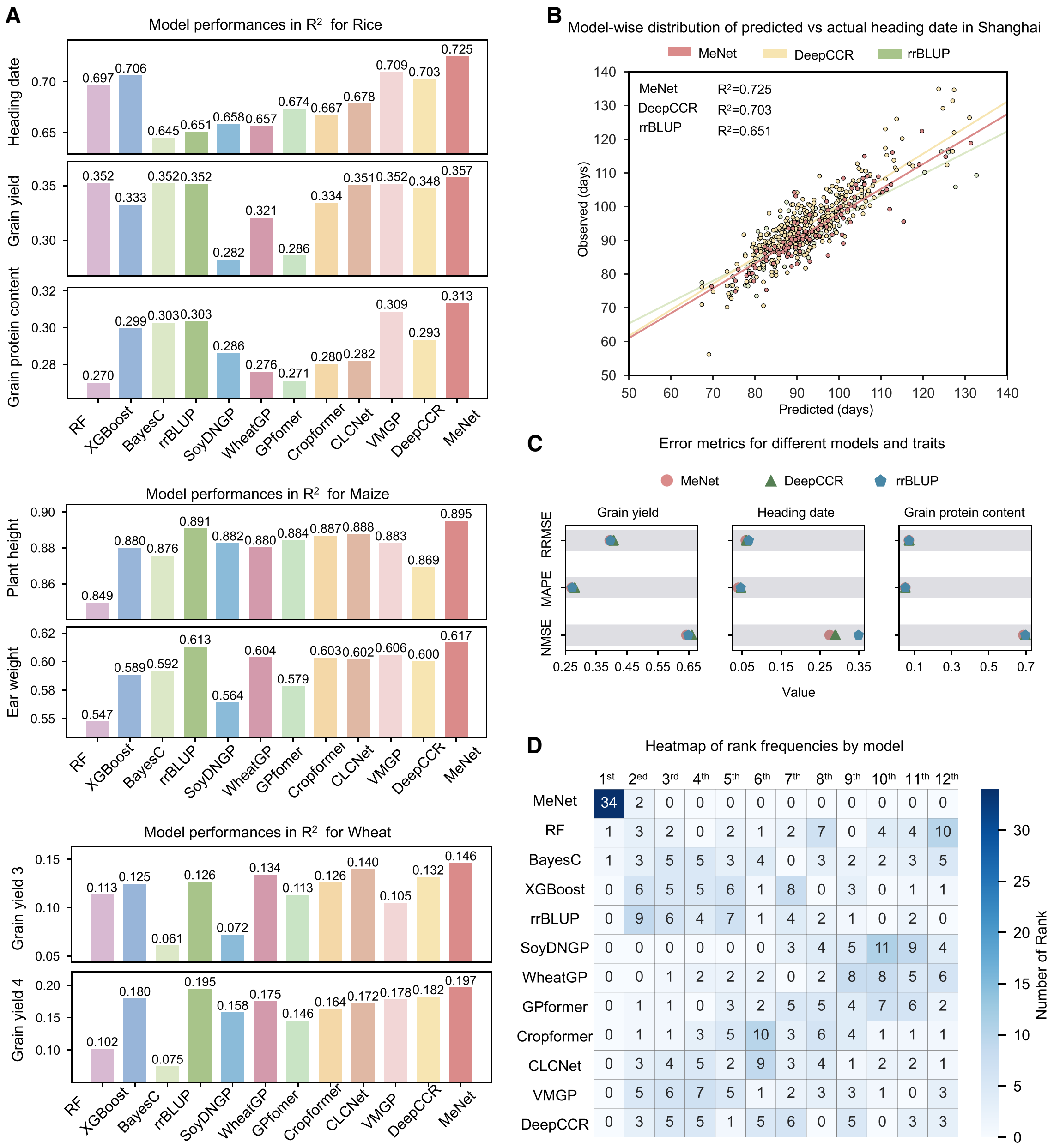

Figure 2. Performance of MeNet on three datasets from three crops.

(A) Bar charts showing the predictive ability (R^2) of MeNet compared with that of RF, XGBoost, BayesC, rrBLUP, SoyDNGP, WheatGP, GPformer, Cropformer, contrastive learning and chromosome-aware network (CLCNet), VMGP, and DeepCCR for rice (Shanghai), wheat, and maize traits with a 6:2:2 split for training, validation, and test sets.

(B) Regression analysis of observed versus predicted HDs in the Shanghai region using MeNet, rrBLUP, and DeepCCR. For simplicity, only 10% of the data points were randomly selected for the plot.

(C) For the Shanghai phenotypic data, comparison of error metrics for MeNet, rrBLUP, and DeepCCR in predicting GY, HD, and GPC.

(D) Statistical plot of predictive performance ranks (R^2) across 36 tests by seven models, shown as a heatmap.

(Gao et al., 2023), WheatGP (Wang et al., 2025b), GPformer (Wu et al., 2023), Cropformer (Wang et al., 2025a), contrastive learning and chromosome-aware network (CLCNet) (Huang et al., 2024), DeepCCR (Ma et al., 2024), and variational auto-encoder-based multi-task genomic prediction (VMGP) (Zhao et al., 2025). These models included two traditional methods, two ML-based approaches, and seven DL-based approaches. All models were trained and tested with a 6:2:2 split for the training, validation, and test sets, with each set showing the same distribution (Supplemental Figure 1). For the 12 rice traits measured in Shanghai, MeNet achieved the highest coefficient of determination (R^2) among all the models (Figure 2A; Supplemental Table 1) and showed a clear advantage in HD prediction compared with the other methods (Figure 2B). For maize, when predicting three key agronomic traits within the same environment, MeNet consistently achieved the highest R^2

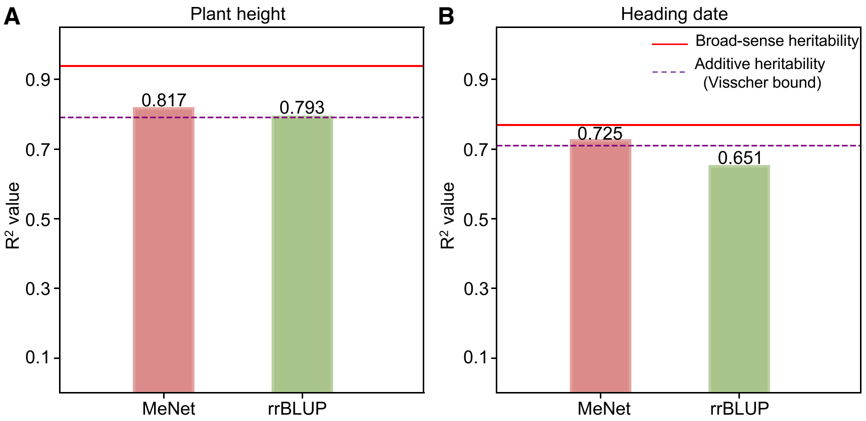

Figure 3. The predictive ability of MeNet demonstrates high efficiency in capturing nonlinearity.
Predictive performance of MeNet and rrBLUP for PH **(A)** and HD **(B)** in Shanghai.

values among all tested methods. For wheat across four different environments, MeNet also delivered the best prediction ability, with the highest R^2 for all GY traits. These results clearly demonstrate the universality and superiority of MeNet for genomic prediction across multiple species, environments, and traits, highlighting its robustness and broad applicability in diverse breeding contexts (Figure 2A; Supplemental Table 1). When the 12 methods were ranked by R^2, MeNet placed first in 34 out of 36 tests and second in the remaining two, whereas the ranks of the other methods varied markedly among different traits or environments (Figure 2D). In fact, it is difficult to determine which model was the second best among the other 11 tested models. The consistent performance of MeNet across multiple phenotypes underscores its robustness and strong adaptability to a wide range of agronomic traits.

We also evaluated other numeric metrics for the rice dataset. MeNet had the highest overall performance in terms of the average Pearson correlation coefficient (PCC) (red lines) and median PCC (blue lines) for all traits in Hangzhou and Hainan (Supplemental Figure 2). MeNet consistently yielded the smallest normalized mean square error, relative root mean square error, and mean absolute percentage error when predicting GY, GPC, and HD (Figure 2C; Supplemental Table 2).

We also tested the prediction ability of MeNet for medium-sized samples. We compared the performance of all models trained and tested using a 3:2:5 split for the training, validation, and test sets; that is, only half of the samples were used for training and parameter optimization. MeNet still showed excellent predictive ability (Supplemental Figures 3 and 4).

Robustness of MeNet for prediction of nonlinear relationships

Robust prediction of nonlinear relatedness is a hallmark of DNNs (Krizhevsky et al., 2017). We investigated the nonlinearity of MeNet for prediction based on the heritability of traits. There are two types of heritability: broad-sense and narrow-sense heritability. Broad-sense heritability (H^2) reflects the overall contribution of genetic factors to phenotypic variance and includes both additive and non-additive effects, whereas narrow-sense heritability (h^2) is confined to additive effects. In a similar manner, the predictive ability of models in terms of R^2 is measured by the proportion of phenotypic variance explained (PVE) by the predicted values of the models. To investigate its role in MeNet's performance, we focused on two traits, PH and HD, measured in Shanghai. The original paper (Wei et al., 2024) estimated that H^2 = 0.938 and h^2 = 0.791 for PH, indicating that approximately 93.8% of the phenotypic variation could be attributed to genetic factors and that approximately 79.1% was additive. For HD, H^2 = 0.767 and h^2 = 0.702. For both traits, non-additive genetic effects contributed significantly to phenotypic variance.

In theory, for an independent test set with independent errors, the prediction metric R^2 of any linear prediction model should not exceed the additive heritability of the trait, h^2, which is sometimes called the "Visscher bound" (Visscher et al., 2008). In the Shanghai rice dataset, this held true for rrBLUP: it achieved an R^2 of 0.793 for PH, which is essentially consistent with the overall additive heritability (Figure 3). Although rrBLUP performed well for this trait, MeNet improved the performance by 3.03%, with a prediction metric R^2 of 0.817, notably exceeding the reported h^2. This suggests that MeNet can effectively capture nonlinear information beyond additive effects. A similar pattern was observed for HD. rrBLUP yielded an R^2 of 0.651, and MeNet achieved an 11.37% improvement, producing an R^2 of 0.725, well above the additive heritability of h^2 = 0.702.

We further analyzed the ability of the model to capture nonlinear responses using locally weighted scatterplot smoothing (LOWESS) (Cleveland and Devlin, 1988). We constructed joint density plots of the observed values and MeNet-predicted values for 12 major phenotypic traits in the Shanghai dataset and overlaid them with LOWESS fitted curves (Supplemental Figure 5). We observed significant bends in our LOWESS curves, indicating the ability of MeNet to capture nonlinearity embedded in the phenotypic data. The plots also revealed some distinct nonlinear patterns. For example, most density plots exhibited an asymmetric "banana-shaped" distribution, suggesting that the model provides higher predictive accuracy in the mid-range values while potentially applying different response mechanisms in the extreme-value regions. MeNet is capable of identifying the nonlinear relationships between genotype and phenotype across different value ranges, highlighting its potential to model complex phenotypic variations. Taken together, these results demonstrate that MeNet is highly effective at capturing nonlinear genetic effects.

Adaptive learning on contributions reflects genetic complexity of quantitative traits in MeNet

We next examined the contribution of genetic background and the effect of variants on trait prediction by MeNet using the integrated gradient (IG) method (Sundararajan et al., 2017) (Figure 4).

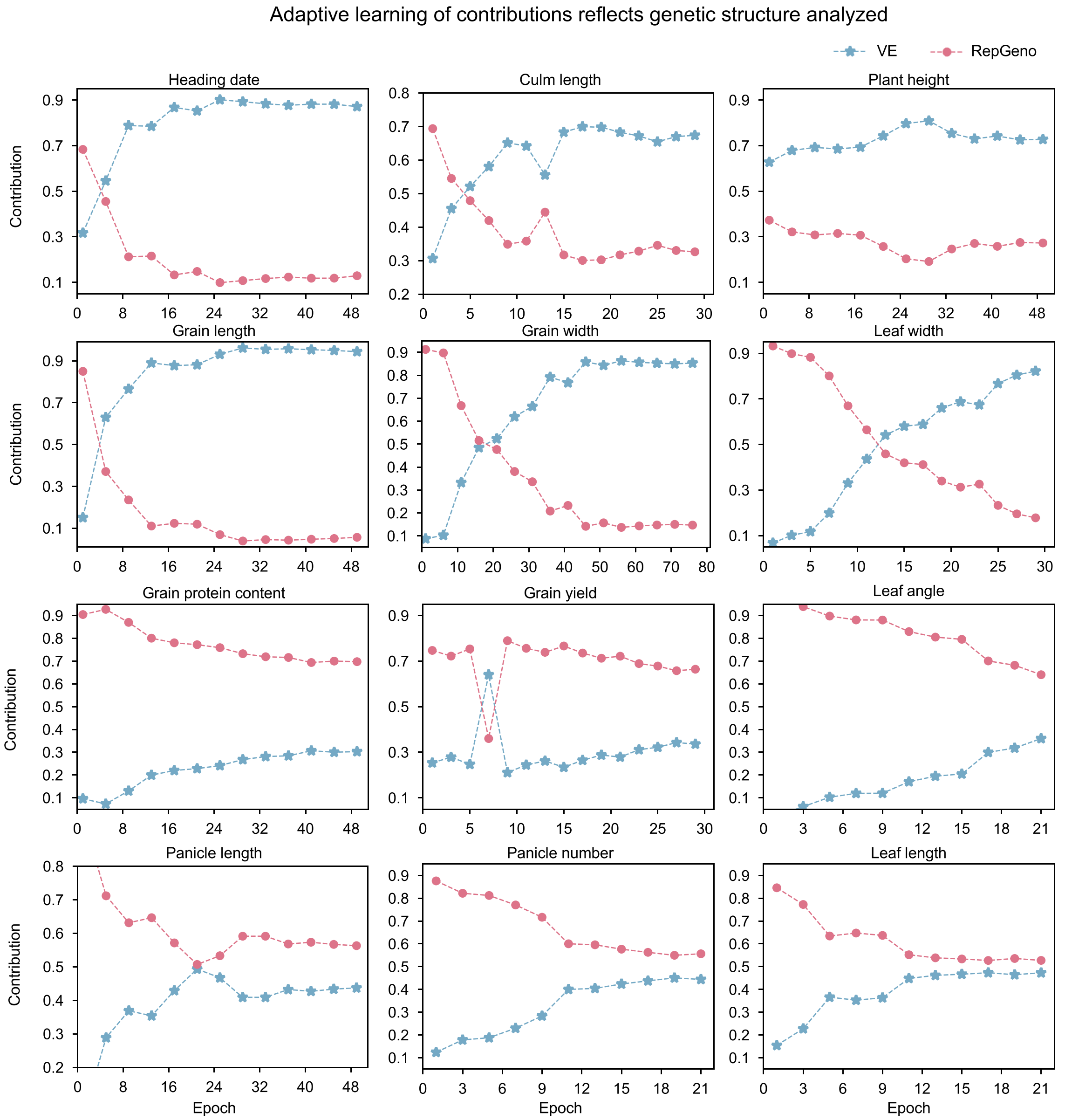

Figure 4. Dynamic patterns of contributions from RepGeno and VE during training for the Shanghai dataset as revealed by IG.
Among 12 agronomic traits, HD, PH, CL, GL, GW, and LW exhibited significantly higher weights for VE (genomic variation information) than for RepGeno (genetic background information). By contrast, for PL, PN, LL, LA, GY, and GPC, the weights of RepGeno were much higher than those of VE.

IG estimates the importance of each input feature by integrating the gradients of the model's output with respect to its inputs along a straight-line path from a baseline input to the actual input. In our model, the final embedding $z = [z_{RepGeno} ; z_{VE}]$ is achieved by combining the genetic relatedness embedding $z_{RepGeno}$ and the variant embedding z_{VE}. We computed the IG of the model output $F(z)$ with respect to z, using a zero vector 0 as the baseline.

Notably, traits with similar genetic architectures exhibited analogous weight-distribution patterns. For example, HD, PH, CL, GL, GW, and LW, which share similar genetic underpinnings, showed nearly identical weight trajectories during training. The final weights of VE (genomic variation information), ranging from 0.70 to 0.90, were significantly higher than those of RepGeno, indicating that the prediction of these traits relies primarily on key genomic variants. This aligns well with our understanding that large-effect genes underlie these traits in the dataset and is consistent with the GWAS results (Supplemental Figure 6).

For traits such as PL, PN, LL, LA, GY, and GPC, RepGeno weights (red lines) were substantially higher than VE weights (blue lines). These traits are, in general, affected by a large number of small-effect genes, as demonstrated by the GWAS results. Instead of directly garnering small effects from a large number of variants dispersed across the whole genome, which would require intensive network training, MeNet leverages genetic background information. Because a large number of small-effect variants lead to a higher correlation with genomic background metrics, such as identity by descent or identity by state, shifting to RepGeno improves predictive reliability and efficiency.

The weight distribution between the genetic background and the cumulative impact of genomic variation in MeNet reflects the genetic complexity of the trait and has important implications for genetic studies and breeding practices. Accordingly, network weights could serve as indicators of a trait's genetic architecture. Furthermore, MeNet's adaptive weighting is highly consistent with breeding practices. For traits such as HD, PH, and CL, where several genes with large effects are predominant, germplasm enhancement could be achieved by introducing genomic variants into key genes or their regulatory elements. For example, precise modulation of the regulatory elements of three genes has been shown to generate rice plants with a broad phenotypic spectrum of HDs (Zhou et al., 2024b). By contrast, for complex traits such as GY and GPC, successful breeding typically involves crossing elite cultivars, followed by intensive selection to accumulate a broader genetic background with an improved combination of small-effect loci. This specific interpretability not only enhances our understanding of trait-prediction mechanisms but also suggests that MeNet is a promising tool for dissecting trait heritability patterns and guiding precision breeding strategies.

The scaling law of genomic variants for prediction

In genomic prediction, the number of variants used for prediction affects not only computational complexity and model performance but also genotyping costs in the field. Previous studies have often suggested feature selection or multivariate regression, such as principal-component regression, to reduce the data dimensions (Long et al., 2011). Although these approaches significantly reduce the computational load and avoid the possibility of overfitting, dimensionality reduction can lead to low performance due to loss of information. Furthermore, there is a general expectation that the model performance of a DNN should improve with increasing quantities of data, a phenomenon termed the scaling law, and data reduction is generally counterproductive for data-driven research. MeNet exploits all genomic variants in a straightforward manner, thus enabling us to investigate the effect of marker number on prediction performance. For simplicity, we applied LD filtering thresholds from 0.1 to 0.5 with an increment of 0.1 to our genomic variants and evaluated the association between marker number and trait performance. In this experimental setting, stricter filtering resulted in fewer markers being used for prediction. The number of SNPs retained under different filtering thresholds is shown in Supplemental Table 3.

In general, when the sample size was large enough, the increase in marker number improved predictive ability, indicating that MeNet aligns well with the scaling of the genomic data. Interestingly, individual traits differed in their response to changes in marker number. For example, when the LD filtering threshold increased from 0.1 to 0.5, the R^2 value for HD rose significantly from 0.541 to 0.725 (33.95%), whereas the $R^{2\ \text{for}}$ GL showed a more moderate increase from 0.617 to 0.726 (17.66%), and the R^2 for GY increased from 0.255 to 0.313 (23.06%).

To identify possible factors contributing to these differences, we examined a frequently used index in breeding—the proportion of PVE by genomic markers. In brief, $PVE = \sigma_g^2 / \left(\sigma_g^2 + \sigma_e^2\right)$, where σ_g^2 is the PVE by markers and σ_e^2 is the PVE by environmental factors. In theory, dimensionality reduction of genomic markers leads to the loss of genomic information, thus reducing the PVE. Indeed, a steady upward trend for PVE was observed as the number of markers increased (Figure 5A). Across 12 traits, PVE and predictive ability were synchronized and exhibited a correlation coefficient ranging from 0.599 to 0.994 (Figure 5B). This strong positive correlation across all traits suggests a direct relationship. Structural equation modeling indicated that PVE was causally linked to predictive ability. The path coefficient from PVE to predictive ability was highly significant ($\beta = 1.04$, $p < 0.001$). These findings confirm that the model's predictive performance is driven primarily by the PVE.

To further explore the effect of marker number on PVE and, subsequently, predictive ability, we subdivided our analysis into two LD-threshold intervals, 0.1–0.3 and 0.3–0.5 (Figure 5C), in the Shanghai dataset. Within the 0.1–0.3 interval, the PVE for 12 traits increased by 2.17%–79.62%, and R^2 increased by 4.60%–25.62%. By contrast, within the 0.3–0.5 range, PVE changed by only −2.00%–2.09%, and R^2 changed by 2.06% to 10.10%. This suggests that in populations with sufficiently large sample sizes, when only a small number of markers are used, predictive ability can be significantly enhanced by increasing marker number, as long as PVE continues to improve. However, this benefit diminishes rapidly as PVE saturates. In addition, the "large *P*, small *N*" problem may increase the risk of overfitting in DL models, offsetting potential gains from increased marker numbers.

Cross-environment prediction with transfer learning

In crop breeding, it is important to breed cultivars with stable yields across multiple environments. This is even more crucial during a time of climate change, when extreme weather occurs frequently. To breed a commercially viable cultivar, breeders need to grow their breeding populations in diverse climates over multiple years for selection. In typical practice, breeders usually own a long-term homesite for crop breeding and rent several commercial test centers where resources are significantly more expensive or difficult to obtain. The rationale of genomic selection (GS) is to save resources by using prediction to reduce the number of plants needed for field testing; thus, reducing the numbers of plants grown at test centers by taking advantage of models trained at the homesite would be highly beneficial and attractive to breeders. This approach requires that the genomic prediction algorithm generalizes well across multiple environments and multiple years.

Here, we investigated the generalization ability of MeNet by extrapolating a model trained for one environment to other environments, using phenotype data from a few samples for training. We used transfer learning, an ML approach that applies prelearned information to a target task, thereby enhancing model performance in limited-data scenarios. We followed a feature-representation transfer paradigm, in which lower-level representations are reused, while higher-level task-specific components are adapted to the new domain. Specifically, the genetic relationship learned from Shanghai using 80% of the samples (60% training and 20% validation) was directly transferred to Hainan and Hangzhou. Fine-tuning was then applied to enhance the model's capacity to capture genetic variation responsive to

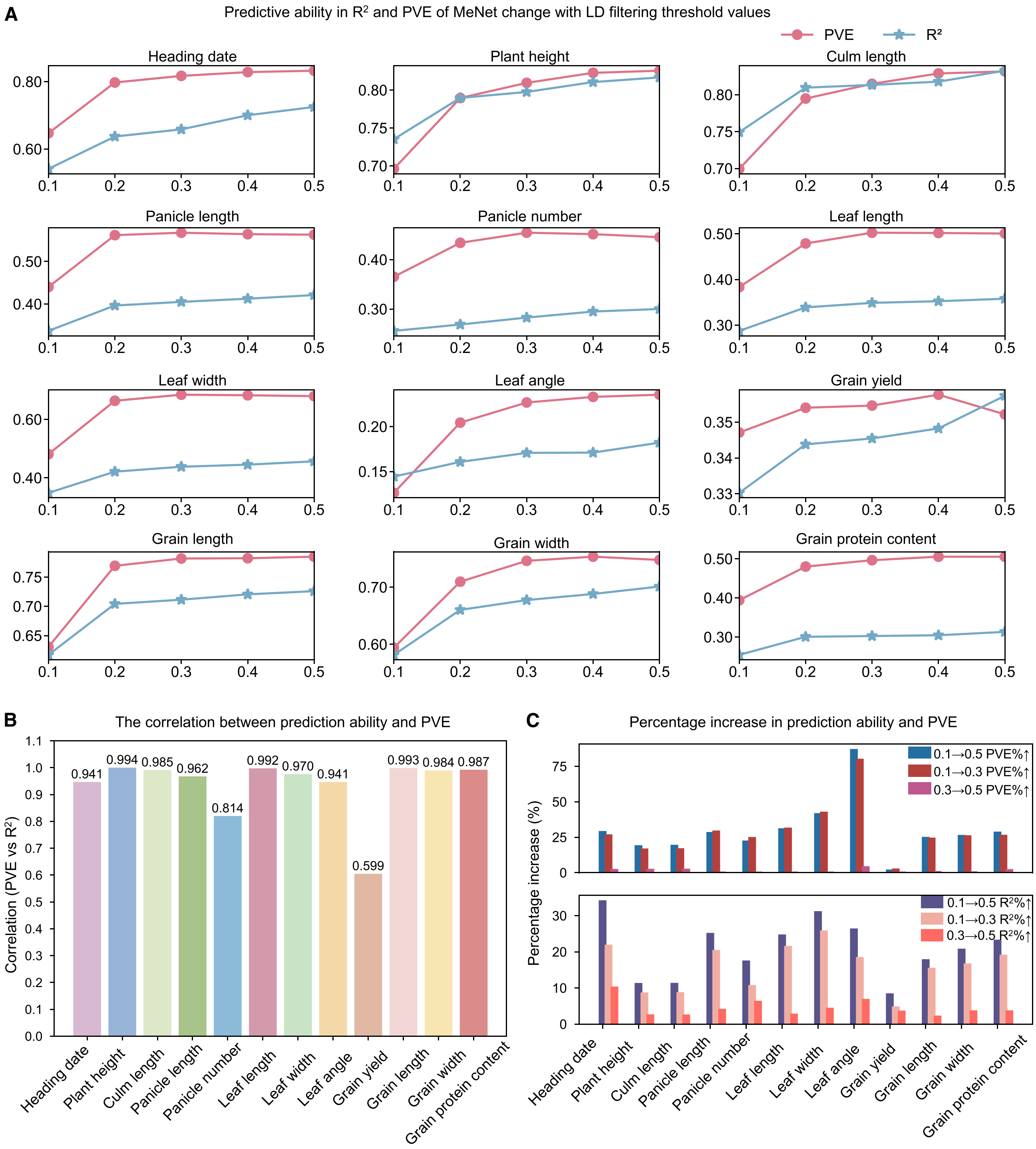

Figure 5. The effect of marker number on predictive ability and PVE for 12 agronomic traits in Shanghai.
(A) The predictive ability (R^2) (blue line) and PVE (red line) increased as LD threshold values changed from 0.1 to 0.5.
(B) Bar chart showing the correlation coefficients between predictive ability and PVE for the 12 traits.
(C) Bar chart showing the percentage increases in predictive ability and PVE across three LD-threshold intervals: 0.1→0.5, 0.1→0.3, and 0.3→0.5.

environment-specific conditions using only 10% of the samples (5% training and 5% validation) from each target environment. This approach facilitates the transfer of stable genetic relatedness modeling while enabling the model to flexibly adapt to the expression of genetic variation under distinct environmental conditions.

For the data from Hangzhou, transfer learning outperformed training from scratch for all traits (Figure 6). Using R^2 as the evaluation metric, the relative advantage of transfer learning ranged from 5.14% to 159.93%. We observed that transfer learning showed a significant advantage for traits with lower prediction accuracy. For instance, the R^2 values for MeNet

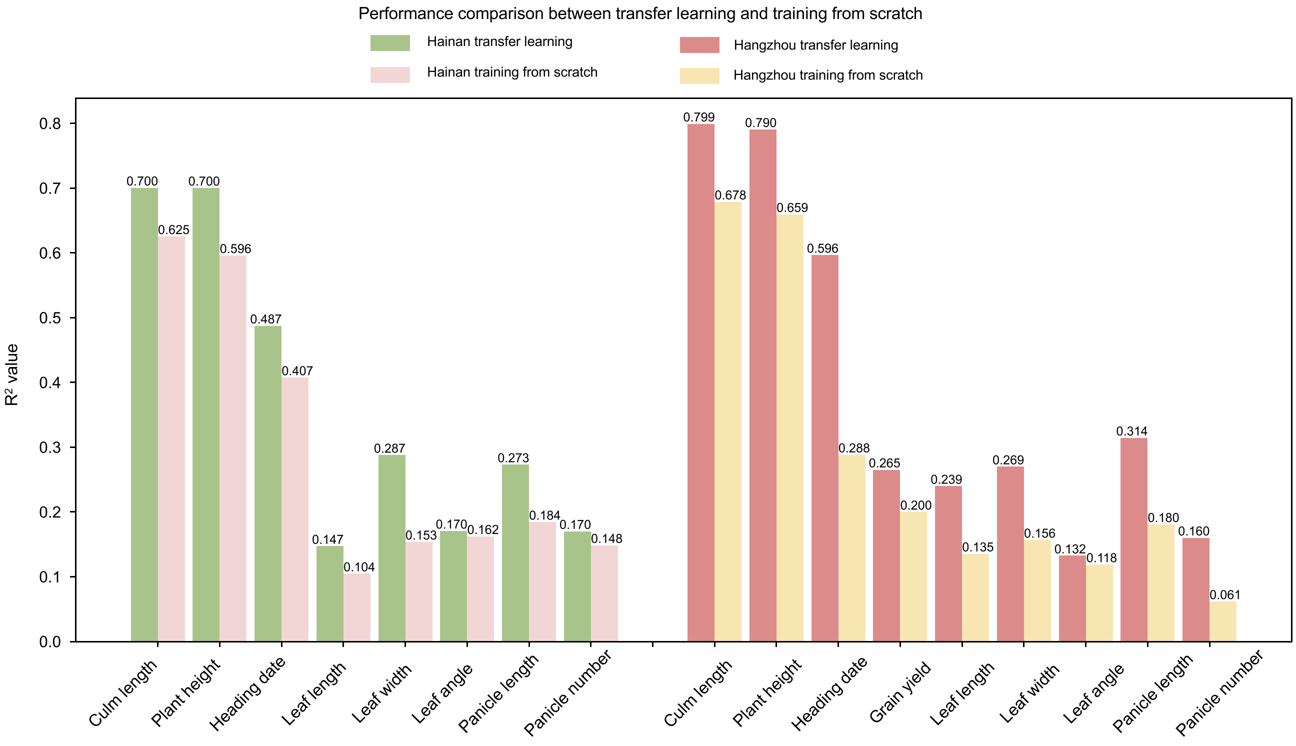

Figure 6. Transfer learning demonstrates the strong ability of MeNet to generalize.
Bar plot of the performance of MeNet under transfer learning and direct training for nine traits in Hangzhou and eight traits in Hainan, using only 10% of the samples.

trained from scratch for PN, HD, and LL were 0.061, 0.288, and 0. 135, respectively, whereas transfer learning yielded R^2 values of 0.160, 0.596, and 0.239, with relative gains of 159.93%, 107. 08%, and 77.29%, respectively. These results suggest that transfer learning shows substantial potential for improving the prediction accuracy of low-precision traits, leading to high stability and preventing model failure. For high-precision traits, transfer learning was still able to significantly improve prediction accuracy. The R^2 for PH when trained from scratch was 0.659, whereas transfer learning resulted in an R^2 of 0.790, a relative gain of 19.89%. For CL, the R^2 when training from scratch was 0.678, and transfer learning increased it to 0.799, a relative gain of 17.73%.

A similar pattern was observed for the data from Hainan. The relative gain of transfer learning ranged from 5.14% to 87.43%. Specifically, for low-precision traits such as LA, LL, and LW, the R^2 values when training from scratch were 0.162, 0.104, and 0. 153, respectively, whereas transfer learning resulted in R^2 values of 0.170, 0.147, and 0.287, corresponding to relative gains of 5. 14%, 40.76%, and 87.43%. These results provide further evidence that transfer learning significantly enhances the prediction accuracy of low-precision traits. For high-precision traits, the R^2 for PH when training from scratch was 0.596, and transfer learning increased it to 0.700, a relative gain of 17.50%. For CL, the R^2 when training from scratch was 0.625, whereas transfer learning yielded an R^2 of 0.700, a relative gain of 12.01%.

These results indicate that transfer learning significantly outperformed training from scratch for all traits, particularly low-precision traits. The MeNet model also increased performance for high-precision traits (e.g., PH), where prediction accuracy was already high. Despite the relatively high precision of these traits, transfer learning still contributed to significant performance enhancement. For low-precision traits (e.g., PN and LA), transfer learning showed more significant improvements, highlighting its importance in ensuring model stability and preventing model failure.

The superior generalization ability of the MeNet model in cross-regional transfer has important implications for practical breeding applications. Theoretically, the focus of genome-wide selection can shift from the development of decentralized proprietary models to large, comprehensive genome-wide selection models. Through collaborations within the field and integration of existing phenotypic and genotypic data, a high-precision foundation model can be trained. For multi-site trait prediction across large populations, the foundation model can be fine-tuned to achieve accurate prediction, reducing the number of field samples and phenotypic data collection efforts, thereby optimizing resource utilization.

DISCUSSION

Continuing and accelerating advances in DL have the potential to transform genomic prediction, but realizing this potential requires a good model design that is compatible with, and even inspiring for, existing biological understanding and breeding practices. Central to such an approach is the integration of genetic background information and the impact of genomic variation—two

key components that correspond to the core breeding strategies of elite cultivar crossing and marker selection. In this study, we introduce MeNet, a mixed-effect DNN that adaptively balances genetic background and the impact of genomic variation, providing a robust approach for genomic prediction. Across multiple traits of three crops in different environments, MeNet significantly outperformed established models such as rrBLUP and VMGP. In addition to its high predictive ability, MeNet offers deeper insights into the genetic architectures of traits through its adaptive learning on contributions from genetic background and the nonlinear cumulative impact of genomic variation. Specifically, the model dynamically adjusts the relative importance of these two sources of information on the basis of the underlying genetic complexity of the trait. By analyzing the final weight distribution, researchers can infer whether a trait is predominantly influenced by several major-effect quantitative trait loci or by numerous smaller-effect variants. This enables precise estimation of genetic complexity, facilitating an informed breeding strategy.

The rationale of GS is to save resources by using prediction to reduce the number of plants needed for field testing; thus, the ability of prediction models to perform well with very few training samples is of crucial significance. In this study, we demonstrated the strong performance of MeNet in extrapolating a well-trained model from one environment to the same trait in other environments by training with phenotypic data from a small percentage of samples. Clearly, extrapolating a well-trained model to the same trait in different years does not pose any challenge. However, MeNet provides a new avenue for GS application in which a well-trained foundation model is established through large, community-wide collaborations and then used to predict multisite, multi-year phenotypes of a large number of samples using data from very few field samples. The code for MeNet has been publicly released, and a shareable software with a graphical user interface is also available (Supplemental Figures 7–10).

Nonetheless, MeNet's high predictive ability comes with costs, and MeNet had the highest computational costs in our experiments. Using HD prediction as an example, the wall time of each epoch for MeNet was 10.136 s, almost 1.5 times that of VMGP (6.705 s) and almost 1.3 times that of DeepCCR (7.567 s) (Supplemental Table 4). This may have been due to its dual-branch architecture, and MeNet could potentially be sped up using parallel computing techniques. In addition, for traits such as HD, whose causal variants are well documented (Wei et al., 2021), prediction with a targeted set of causal variants or with related metabolite or molecular markers (Xu et al., 2025) instead of genome-wide markers could be computationally efficient and biologically interpretable. This approach is regularly practiced for specific traits in molecular breeding and could be a potential alternative GS model for balancing accuracy and resource efficiency (Kitazawa et al., 2024), albeit only for a few well-studied traits.

A key advantage of the DNN is its ability to detect nonlinear relationships among genomic variants. Our analysis of PH demonstrates the capacity of MeNet to capture complex interactions, including gene-by-gene and potential gene-by-environment interactions. This highlights its broader potential for dissection of intricate genetic architectures in which nonlinear effects play a crucial role. Nonetheless, pinpointing the exact gene-by-gene or gene-by-environment interactions and constructing gene interaction networks remains an open challenge that requires specialized interpretability techniques. Confirming the ability of MeNet to accurately capture epistasis and gene-by-environment interactions requires further investigation using larger or multi-environment datasets. Such studies will be essential to fully harness its capabilities for breeding practice and genomic research.

METHODS

Data preprocessing

Genomic predictions used the 18000 rice panel (n = 18 421 accessions), which includes 15 recombinant inbred line populations and a four-way multiparent advanced generation inter-cross population (Wei et al., 2024). Phenotypes for agronomic traits were collected across three environments (n = 18 421 per environment) for the populations. The design of the populations, characterized by a relatively weak population structure, facilitates clearer phenotypic differentiation among samples. In general, the integrative nature and reliability of these data provide a robust foundation for advancing our understanding of the genetic architecture of rice and serve as a valuable resource for genomic prediction studies. The phenotypic data included 12 numeric agronomic traits: CL, PH, HD, GY, GL, GW, GPC, LL, LW, LA, PL, and PN. All traits showed a normal distribution, and no additional transformations were applied.

To ensure data integrity and analytical reliability, a comprehensive quality control (QC) workflow was implemented using PLINK (v1.9). The QC workflow comprised two main steps: (1) variant filtering on the basis of minor allele frequency, in which genomic markers with a minor allele frequency <0.01 were excluded, and (2) LD filtering, in which a sliding window was used to exclude markers that exhibited high LD. The window size was set to 2000 bp, with a step size of 1000 bp. LD thresholds (r^2) from 0.1 to 0.5 were tested to evaluate the influence of LD on prediction performance.

For maize, the dataset was constructed using 30 recombinant inbred line F1 populations and 1428 parental lines derived from the complete diallel used for breeding and intercross (CUBIC) collection, resulting in a total of 42 480 hybrid combinations (Xiao et al., 2021). Field phenotyping was performed on 8652 F1 hybrids to assess three key agronomic traits: days to tasseling, PH, and ear weight. Data preprocessing with PLINK involved LD filtering using –indep-pairwise 1000 100 0.1, resulting in 30 890 SNPs. After handling missing values, 8153 individuals were retained, and all seven models were tested. For wheat, the dataset contained GY data from four different environments (GY1, GY2, GY3, and GY4) with 599 samples and 1279 features (Crossa et al., 2010).

After QC, the genomic variants were encoded with 1 and −1 for homozygous alleles and 0 for heterozygous alleles. Missing genotype values were imputed with 0.

Implementation of MeNet

MeNet uses a dual-branch architecture. The first branch is inspired by random effects in LMMs. We proposed a novel neural network to produce an embedding as the representation of genetic-relatedness information among samples (referred to as RepGeno). We then designed a convolutional neural network enhanced with residual units to serve as the second branch, called the VE module. The VE module uses a convolutional network to extract regional dependencies and long-range interactions across loci. These two representations are aligned and fused by the fusion module, whose core is a cross-information feature fusion unit (Figure 1C), and MeNet dynamically adjusts their contributions on the basis of adaptive learning of the genetic architecture of the target phenotype.

We performed ablation experiments on the two branches of MeNet and validated their effectiveness (Supplemental Table 5).

Representation learning of a population via trait-aware contrastive learning

Inspired by triplet contrastive learning and the Siamese network, we developed RepGeno, a phenotype-aware genotypic representation learning framework for modeling trait-specific genetic relationships. The objective of RepGeno is to learn an embedding space that simultaneously captures population-level classification and individual-level phenotypic continuity, thereby enabling the computation of genetic relationships that reflect both population affiliation and trait similarity among samples. In other words, RepGeno aims to constrain the embeddings of samples from different populations such that their distances correspond to phenotypic differences. RepGeno (Figure 1B) uses trait-aware triplet contrastive learning to train a trait-specific encoder, which maps each sample's genotype into an embedding vector that captures both population-level differences and individual phenotypic variations. Subsequently, by calculating the genetic distances between samples, RepGeno produces an embedding that represents the genetic relatedness among samples.

Each sample is treated as an anchor, with a positive sample randomly drawn from the same population and a negative sample randomly drawn from a different population. We designed a trait-aware triplet loss function, in which the margin is adaptively scaled according to the phenotypic differences between the anchor and the positive/negative samples. This loss encourages the distance between positive and negative pairs to align with their trait dissimilarities, thereby achieving inter-population separation and intra-population phenotypic continuity modeling. The trait-aware triplet loss is formally defined as follows:

$$\mathcal{L} = \log\left(1 + e^{\left(d^{+} - d^{-} + m_{\gamma}\right)}\right) \text{ and}$$

$$m_{\gamma} = \frac{\Delta^{-}}{\Delta^{+} + \varepsilon} \times margin.$$

d^{+} and d^{-} denote the Euclidean distances between the normalized embedding vectors of the positive and negative sample pairs, respectively. Δ^{-} and Δ^{+} represent the absolute phenotypic differences between the positive and negative sample pairs, respectively. When population affiliation information is unavailable in the dataset, for each anchor, we randomly sample two other samples and determine whether they serve as positive or negative samples on the basis of their phenotypic differences.

The genetic similarity between samples is represented by the normalized Euclidean distance (scaled to the range [0, 1]) between their embeddings in the optimized latent space. This learned relatedness offers a task-sensitive alternative to traditional genetic relationships and enables more adaptive correction in trait-specific genomic analyses. Note that the genetic relatedness distances extracted by RepGeno to produce the final embedding are quite different from the genetic relatedness matrix used in LMMs, as RepGeno takes into account the phenotypic values.

Modeling genotypic variation and information fusion

In parallel, VE extracts information on genetic variants into embedding Z_{VE} via a convolutional layer, two residual blocks based on ResNet architecture, followed by an adaptive average pooling layer and fully connected layers.

In the message-passing process of MeNet, $Z_{RepGeno}$ and Z_{VE} are regarded as two information vectors. Inspired by cross-view feature fusion (Ye et al., 2024), we developed cross-information feature fusion to enable efficient interaction between these vectors while preserving their respective structural characteristics. Specifically, the concatenated joint information from the two vectors is fed into two independent correction networks, $\mathcal{F}_{VE}$ and $\mathcal{F}_{RepGeno}$, to generate correction vectors, which are then fed back to the original information through a residual mechanism.

$$\widehat{Z_{VE}} = Z_{VE} + \mathcal{F}_{VE}\left(\left[Z_{VE}; Z_{RepGeno}\right]\right)$$

$$\widehat{Z_{RepGeno}} = Z_{RepGeno} + \mathcal{F}_{RepGeno}\left(\left[Z_{VE}; Z_{RepGeno}\right]\right)$$

$\mathcal{F}_{VE}$ and $\mathcal{F}_{RepGeno}$ share the same bottleneck architecture composed of three fully connected layers but are trained independently to ensure that both information flows follow an identical correction paradigm. The corrected information flows are then concatenated again and passed through three fully connected layers to produce the final prediction output.

Prediction across diverse environments

To enable MeNet to generalize across environments, we adopted a parameter-efficient transfer learning approach, in which most parts of the model remain intact and only specific parts are fine-tuned. This strategy is particularly popular in DL when limited labeled data in the target domain are available. After comprehensive training of MeNet using data from one environment, the RepGeno-based encoder and its associated feature extractor are frozen and transferred to a target environment. The fusion module, including $\mathcal{F}_{VE}$ of cross-information feature fusion and the three-layer fully connected network used for the final prediction output, is fine-tuned using the data from the target environment.

Chromosome-aware window-encoding mechanism

To address redundancy and parameter explosion, we introduced a chromosome-aware window-encoding mechanism. Instead of processing the entire genotype vector with a single shared network, we first divide the genotype by chromosome. For each chromosome-specific segment, we assign an independent VE to capture localized genomic patterns. The resulting chromosome-wise embeddings are then integrated through an extended cross-information feature fusion module for multi-source information interaction, producing the final embedding representation for downstream modeling. This design significantly reduces computational load and improves parameter efficiency by introducing modular and biologically informed encoding.

Training of models

RepGeno and MeNet were trained on NVIDIA A800 GPUs (80 GB memory) and optimized using the AdamW optimizer with weight-decay regularization. The mean absolute error was adopted as the training objective. Training was conducted in mini-batches of size 128 with randomized data loading to promote stable convergence. In RepGeno, the distance between an anchor and its negative counterpart should exceed that between the anchor and its positive counterpart by at least a margin value, and the margin parameter space was set to [0.1, 0.2, 0.3, 0.4, 0.5]. For VE, the number of convolutional kernel channels was searched within [1, 2, 4, 8, 16, 64]. For VE and RepGeno, the embedding dimension was explored in [512, 1024, 4096, 8192] (Supplemental Table 6). Learning rates were dynamically adjusted on the basis of validation performance and reduced by a factor when the validation metric plateaued for several epochs. The best-performing checkpoints were selected on the basis of the validation dataset R^2 and used for the final evaluation on the test dataset.

We selected 11 representative traditional and modern ML models for comparison: RF, XGBoost, BayesC, rrBLUP, SoyDNGP, WheatGP, GPformer, Cropformer, contrastive learning and chromosome-aware network, DeepCCR, and VMGP. GPformer and DeepCCR, for which neither the source code nor the executable software is publicly available, were implemented according to their publications. All models were trained and evaluated using the same settings (6:2:2, 3:2:5, and 0.5:0.5:9 for training, validation, and test sets). Hyperparameter optimization for each model was conducted on the validation set in terms of R^2, PCC, and mean absolute error metrics.

Evaluating the contributions from RepGeno and VE embeddings via Integrated Gradients (IG)

To gain a deeper understanding of how MeNet utilizes information from the RepGeno and VE modules, we used IG as a post hoc interpretability method. IG quantifies feature importance by averaging gradients along a linear path from a baseline to the input. Given a differentiable prediction function F, an input sample x, and a baseline input x', representing the absence of a signal, the attribution for the i-th feature is computed as

$$IG_i(x) = (x_i - x_i') \int_0^1 \frac{\partial F(x_i' + \alpha(x - x'))}{\partial x_i} d\alpha.$$

This formulation integrates the gradient of the output along the path from x' to x, capturing the marginal contribution of each input dimension in a path-aware manner.

The fused embedding $z = [z_{VE}; z_{RepGeno}]$ is used to compute the IG. For a dataset of N samples, let $a_j = IG(z_j)$ denote the attribution vector for sample j, where i denotes the index of the attribution vector. We compute the average absolute attribution across samples:

$$S_{VE} = \sum_{i \in VE} \left(\frac{1}{N} \sum_{j=1}^{N} |a_{j,i}| \right) \text{ and}$$

$$S_{RepGeno} = \sum_{i \in RepGeno} \left(\frac{1}{N} \sum_{j=1}^{N} |a_{j,i}| \right).$$

The scalar values S_{VE} and $S_{RepGeno}$ represent the overall attribution assigned to the variant-level and population-level components, respectively. Optionally, we normalize these scores to obtain relative contributions by computing their proportions with respect to the total attribution sum.

DATA AND CODE AVAILABILITY

The rice phenotypic data used in this study are available in the online supplementary materials of Wei et al. (2024). The genomic data used in this study can be downloaded from https://figshare.com/s/12978737918eecb74903. For maize, we used the Maize8652 dataset (Xiao et al., 2021), which is accessible at https://iagr.genomics.cn/CropGS/#/Datasets. For wheat, we used a publicly available dataset (Crossa et al., 2010), available at https://academic.oup.com/genetics/article/186/2/713/6063582#supplementary-data.

The source code for MeNet is available on GitHub at https://github.com/ganlab/MENET. An executable software tool with a graphical user interface for the Windows operating system can be downloaded from https://github.com/ganlab/menet/releases.

FUNDING

This work was supported in part by grants from the National Science and Technology Major Project, China (grant no. 2022ZD0115703); the National Natural Science Foundation of China (grant no. 32176047); the National Science Foundation of Jiangsu Province (grant no. BE2022383); the Jiangsu Engineering Research Center for Plant Genome Editing; Southern Japonica Rice Research and Development Co., Ltd.; and the Jiangsu Collaborative Innovation Center for Modern Crop Production, China.

ACKNOWLEDGMENTS

We thank Professor Miltos Tsiantis for his valuable suggestions and Mr. Jun Wang and Mr. Ziya Tang for their technical assistance. No conflict of interest declared.

AUTHOR CONTRIBUTIONS

X.G. and J.W. conceived and supervised the study and revised the manuscript. Y.L. and S.R. performed the data analysis, model development, and evaluation and wrote the manuscript. J. Li and J. Lee analyzed the data. All authors reviewed and approved the final manuscript.

SUPPLEMENTAL INFORMATION

Supplemental information is available at *Plant Communications Online*.

Received: July 23, 2025
Revised: October 17, 2025
Accepted: November 17, 2025
Published: November 19, 2025

REFERENCES

Abdollahi-Arpanahi, R, Gianola, D, and Peñagaricano, F (2020). Deep learning versus parametric and ensemble methods for genomic prediction of complex phenotypes. Genet Sel. Evol **52**:12. https://doi.org/10.1186/s12711-020-00531-z.

Cheverud, J.M., and Routman, E.J. (1995). Epistasis and its contribution to genetic variance components. Genetics **139**:1455–1461.

Cheverud, J.M., Vaughn, T.T., Pletscher, L.S., King-Ellison, K., Bailiff, J., Adams, E., Erickson, C., and Bonislawski, A. (1999). Epistasis and the Evolution of Additive Genetic Variance in Populations That Pass Through a Bottleneck. Evolution **53**:1009–1018.

Cleveland, W.S., and Devlin, S.J. (1988). Locally Weighted Regression: An Approach to Regression Analysis by Local Fitting. J. Am. Stat. Assoc. **83**:596–610.

Crossa, J., Campos, G.D.L., Pérez, P., Gianola, D., Burgueño, J., Araus, J.L., Makumbi, D., Singh, R.P., Dreisigacker, S., Yan, J., et al. (2010). Prediction of Genetic Values of Quantitative Traits in Plant Breeding Using Pedigree and Molecular Markers. Genetics **186**:713–724.

Dalla-Torre, H., Gonzalez, L., Mendoza-Revilla, J., et al. (2025). Nucleotide Transformer: building and evaluating robust foundation models for human genomics. Nat Methods **22**:287–297. https://doi.org/10.1038/s41592-024-02523-z.

De Los Campos, G., Naya, H., Gianola, D., Crossa, J., Legarra, A., Manfredi, E., Weigel, K., and Cotes, J.M. (2009). Predicting Quantitative Traits With Regression Models for Dense Molecular Markers and Pedigree. Genetics **182**:375–385.

Du, C., Wei, J., Wang, S., and Jia, Z. (2018). Genomic selection using principal component regression. Heredity **121**:12–23.

Gao, P., Zhao, H., Luo, Z., Lin, Y., Feng, W., Li, Y., Kong, F., Li, X., Fang, C., and Wang, X. (2023). SoyDNGP: a web-accessible deep learning framework for genomic prediction in soybean breeding. Brief. Bioinform. **24**:bbad349.

Gianola, D. (2013). Priors in Whole-Genome Regression: The Bayesian Alphabet Returns. Genetics **194**:573–596.

Gianola, D., De Los Campos, G., Hill, W.G., Manfredi, E., and Fernando, R. (2009). Additive Genetic Variability and the Bayesian Alphabet. Genetics **183**:347–363.

Hayes, B., and Goddard, M. (2010). Genome-wide association and genomic selection in animal breeding. Genome **53**:876–883.

Hollmann, N., Müller, S., Purucker, L., Krishnakumar, A., Körfer, M., Hoo, S.B., Schirrmeister, R.T., and Hutter, F. (2025). Accurate predictions on small data with a tabular foundation model. Nature **637**:319–326.

Huang, J., Yang, Z., Yin, M., Li, C., Li, J., Wang, Y., Huang, L., Liang, C., He, F., Li, M., et al. (2024). CLCNet: a contrastive learning and chromosome-aware network for genomic prediction in plants. Preprint at bioRxiv. https://doi.org/10.1101/2024.12.29.630569.

Ji, Y., Zhou, Z., Liu, H., and Davuluri, R.V. (2021). DNABERT: pre-trained Bidirectional Encoder Representations from Transformers model for DNA-language in genome. Bioinformatics **37**:2112–2120.

Kawakita, S., Yamasaki, M., Teratani, R., Yabe, S., Kajiya-Kanegae, H., Yoshida, H., Fushimi, E., and Nakagawa, H. (2024). Dual ensemble approach to predict rice heading date by integrating multiple rice phenology models and machine learning-based genetic parameter regression models. Agric. For. Meteorol. **344**:109821.

Kitazawa, N., Shomura, A., Mizubayashi, T., Ando, T., Hayashi, N., Yabe, S., Matsubara, K., Ebana, K., Yamanouchi, U., and Fukuoka, S. (2024). Development of SNP genotyping assays for heading date in rice. Breed. Sci. **74**:274–284.

Krizhevsky, A., Sutskever, I., and Hinton, G.E. (2017). ImageNet classification with deep convolutional neural networks. Commun. ACM **60**:84–90.

Long, N., Gianola, D., Rosa, G.J.M., and Weigel, K.A. (2011). Dimension reduction and variable selection for genomic selection: application to predicting milk yield in Holsteins. J. Anim. Breed. Genet. **128**:247–257.

Ma, X., Wang, H., Wu, S., Han, B., Cui, D., Liu, J., Zhang, Q., Xia, X., Song, P., Tang, C., et al. (2024). DeepCCR : large-scale genomics-based deep learning method for improving rice breeding. Plant Biotechnol. J. **22**:2691–2693.

Mackay, T.F.C. (2001). The Genetic Architecture of Quantitative Traits. Annu. Rev. Genet. **35**:303–339.

Meher, P.K., Rustgi, S., and Kumar, A. (2022). Performance of Bayesian and BLUP alphabets for genomic prediction: analysis, comparison and results. Heredity **128**:519–530.

Meuwissen, T.H., Hayes, B.J., and Goddard, M.E. (2001). Prediction of Total Genetic Value Using Genome-Wide Dense Marker Maps. Genetics **157**:1819–1829.

Montesinos López, O.A., Montesinos López, A., and Crossa, J. (2022). Random Forest for Genomic Prediction. In Multivariate Statistical Machine Learning Methods for Genomic Prediction, O.A. Montesinos López, A. Montesinos López, and J. Crossa, eds. (Springer International Publishing), pp. 633–681.

Ogutu, J.O., Piepho, H.-P., and Schulz-Streeck, T. (2011). A comparison of random forests, boosting and support vector machines for genomic selection. BMC Proc. **5**:S11.

Okut, H. (2021). Deep Learning Algorithms for Complex Traits Genomic Prediction. Hayvan Bilimi Ve Ürün. Derg. **4**:225–239.

Rogers, A.R., Dunne, J.C., Romay, C., Bohn, M., Buckler, E.S., Ciampitti, I.A., Edwards, J., Ertl, D., Flint-Garcia, S., Gore, M.A., et al. (2021). The importance of dominance and genotype-by-environment interactions on grain yield variation in a large-scale public cooperative maize experiment. G3 (Bethesda). **11**:jkaa050.

Spindel, J., Begum, H., Akdemir, D., Virk, P., Collard, B., Redoña, E., Atlin, G., Jannink, J.-L., and McCouch, S.R. (2015). Genomic Selection and Association Mapping in Rice (Oryza sativa): Effect of Trait Genetic Architecture, Training Population Composition, Marker Number and Statistical Model on Accuracy of Rice Genomic Selection in Elite, Tropical Rice Breeding Lines. PLoS Genet. **11**:e1004982.

Sundararajan, M., Taly, A., and Yan, Q. (2017). Axiomatic Attribution for Deep Networks. In Proceedings of the 34th International Conference on Machine Learning (PMLR), pp. 3319–3328.

The Million Veteran Program, Evangelou, E., Warren, H.R., Mosen-Ansorena, D., Mifsud, B., Pazoki, R., Gao, H., Ntritsos, G., Dimou, N., Cabrera, C.P., et al. (2018). Genetic analysis of over 1 million people identifies 535 new loci associated with blood pressure traits. Nat. Genet. **50**:1412–1425.

Tibshirani, R. (1996). Regression Shrinkage and Selection via the Lasso. J. R. Stat. Soc. Ser. B Methodol. **58**:267–288.

Visscher, P.M., Hill, W.G., and Wray, N.R. (2008). Heritability in the genomics era — concepts and misconceptions. Nat. Rev. Genet. **9**:255–266.

Wang, D., Li, J., Wang, Y., and Wang, E. (2022). A comparison on predicting functional impact of genomic variants. NAR Genom. Bioinform. **4**:lqab122.

Wang, K., Abid, M.A., Rasheed, A., Crossa, J., Hearne, S., and Li, H. (2023). DNNGP, a deep neural network-based method for genomic prediction using multi-omics data in plants. Mol. Plant **16**:279–293.

Wang, H., Yan, S., Wang, W., Chen, Y., Hong, J., He, Q., Diao, X., Lin, Y., Chen, Y., Cao, Y., et al. (2025a). Cropformer: An interpretable deep learning framework for crop genomic prediction. Plant Commun. **6**:101223.

Wang, C., Zhang, D., Ma, Y., Zhao, Y., Liu, P., and Li, X. (2025b). WheatGP, a genomic prediction method based on CNN and LSTM. Brief. Bioinform. **26**:bbaf191.

Wei, X., Qiu, J., Yong, K., Fan, J., Zhang, Q., Hua, H., Liu, J., Wang, Q., Olsen, K.M., Han, B., and Huang, X. (2021). A quantitative genomics map of rice provides genetic insights and guides breeding. Nat. Genet. **53**:243–253.

Wei, X., Chen, M., Zhang, Q., Gong, J., Liu, J., Yong, K., Wang, Q., Fan, J., Chen, S., Hua, H., et al. (2024). Genomic investigation of 18,421 lines reveals the genetic architecture of rice. Science **385**:eadm8762.

Wu, C., Zhang, Y., Ying, Z., Li, L., Wang, J., Yu, H., Zhang, M., Feng, X., Wei, X., and Xu, X. (2023). A transformer-based genomic prediction method fused with knowledge-guided module. Brief. Bioinform. **25**:bbad438.

Xiang, Y., Xia, C., Li, L., Wei, R., Rong, T., Liu, H., and Lan, H. (2024). Genomic prediction of yield-related traits and genome-based establishment of heterotic pattern in maize hybrid breeding of Southwest China. Front. Plant Sci. **15**:1441555.

Xiao, Y., Jiang, S., Cheng, Q., Wang, X., Yan, J., Zhang, R., Qiao, F., Ma, C., Luo, J., Li, W., et al. (2021). The genetic mechanism of heterosis utilization in maize improvement. Genome Biol. **22**:148.

Xu, Y., Yang, W., Qiu, J., Zhou, K., Yu, G., Zhang, Y., Wang, X., Jiao, Y., Wang, X., Hu, S., et al. (2025). Metabolic marker-assisted genomic prediction improves hybrid breeding. Plant Commun. **6**:101199.

Yan, J., Xu, Y., Cheng, Q., Jiang, S., Wang, Q., Xiao, Y., Ma, C., Yan, J., and Wang, X. (2021). LightGBM: accelerated genomically designed crop breeding through ensemble learning. Genome Biol. **22**:271.

Ye, J., Luo, Q., Yu, J., Zhong, H., Zheng, Z., He, C., and Li, W. (2024). SG-BEV: Satellite-Guided BEV Fusion for Cross-View Semantic Segmentation. Preprint at arXiv. https://doi.org/10.48550/arXiv.2404.02638.

Zhang, B., Liu, H., Qi, F., Zhang, Z., Li, Q., Han, Z., and Xing, Y. (2019). Genetic Interactions Among Ghd7, Ghd8, OsPRR37 and Hd1 Contribute to Large Variation in Heading Date in Rice. Rice **12**:48.

Zhao, X., Sun, F., Li, J., Zhang, D., Zhang, Q., Liu, Z., Tan, C., Ma, H., and Wang, K. (2025). VMGP: A unified variational auto-encoder based multi-task model for multi-phenotype, multi-environment, and cross-population genomic selection in plants. Artif. Intell. Agric. **15**:829–842.

Zhou, Z., Ji, Y., Li, W., Dutta, P., Davuluri, R., and Liu, H. (2024a). DNABERT-2: Efficient Foundation Model and Benchmark For Multi-Species Genome. Preprint at arXiv. https://doi.org/10.48550/arXiv.2306.15006.

Zhou, S., Cai, L., Wu, H., Wang, B., Gu, B., Cui, S., Huang, X., Xu, Z., Hao, B., Hou, H., et al. (2024b). Fine-tuning rice heading date through multiplex editing of the regulatory regions of key genes by CRISPR-Cas9. Plant Biotechnol. J. **22**:751–758.

Zou, J., Huss, M., Abid, A., Mohammadi, P., Torkamani, A., and Telenti, A. (2019). A primer on deep learning in genomics. Nat. Genet. **51**:12–18.

Plant Communications, Volume 7

Supplemental information

MeNet: A mixed-effect deep neural network for multi-environment genomic prediction of agronomic traits

Yanhui Li, Shengjie Ren, Jixiang Li, Jiyong Lee, Jianmin Wan, and Xiangchao Gan

Supplementary information

Mixed-effect deep neural network for multi-environment genomic prediction of agronomic traits

Yanhui Li[1†], Shengjie Ren[1†], Jixiang Li[1], Jiyong Lee[1], Jianmin Wan[2,3*], Xiangchao Gan[1,2*]

[1]State Key Laboratory for Crop Genetics and Germplasm Enhancement and Utilization, Jiangsu Nanjing National Field Scientific Observation and Research Station for Rice Germplasm, Key Laboratory of Biology, Genetics and Breeding of Japonica Rice in Mid-lower Yangtze River, Ministry of Agriculture and Rural Affairs, Academy for Advanced Interdisciplinary Studies, Nanjing Agricultural University, Nanjing 210095, China.

[2]Zhongshan Biological Breeding Laboratory, Nanjing 210095, China.

[3]State Key Laboratory of Crop Gene Resources and Breeding, Institute of Crop Sciences, Chinese Academy of Agricultural Sciences, Beijing, China.

Supplementary Table 1| Prediction performance (R^2) of 11 models across 36 tests, including 29 rice traits evaluated under three environments, three maize traits, and wheat GY evaluated under four environments.

	Phenotype	RF	XGBoost	BayesC	rrBLUP	SoyDNGP	WheatGP	GPformer	Cropformer	CLCNet	VMGP	DeepCCR	MeNet
Rice for Shanghai	CL	0.802	0.828	0.805	0.803	0.803	0.802	0.795	0.803	0.804	0.812	0.814	0.833
	GL	0.645	0.713	0.711	0.726	0.676	0.669	0.684	0.688	0.692	0.721	0.721	0.726
	GW	0.630	0.686	0.677	0.686	0.621	0.629	0.630	0.599	0.593	0.693	0.688	0.701
	GY	0.352	0.333	0.352	0.352	0.282	0.321	0.286	0.334	0.351	0.352	0.348	0.357
	HD	0.697	0.706	0.645	0.651	0.658	0.657	0.674	0.667	0.678	0.709	0.703	0.725
	LA	0.173	0.174	0.169	0.172	0.171	0.167	0.175	0.182	0.177	0.176	0.178	0.182
	LL	0.327	0.347	0.353	0.353	0.303	0.297	0.297	0.341	0.348	0.337	0.308	0.358
	LW	0.365	0.435	0.442	0.443	0.394	0.399	0.379	0.425	0.388	0.443	0.420	0.457
	PL	0.386	0.414	0.414	0.411	0.384	0.375	0.399	0.393	0.397	0.404	0.402	0.421
	PN	0.267	0.282	0.299	0.297	0.274	0.287	0.275	0.291	0.298	0.279	0.263	0.300
	PH	0.795	0.809	0.792	0.793	0.786	0.785	0.789	0.792	0.792	0.796	0.809	0.817
	GPC	0.270	0.300	0.303	0.303	0.286	0.276	0.271	0.280	0.282	0.309	0.293	0.313
Rice for Hainan	CL	0.743	0.769	0.760	0.760	0.745	0.750	0.753	0.752	0.763	0.765	0.759	0.772
	HD	0.643	0.658	0.636	0.639	0.637	0.645	0.650	0.633	0.635	0.641	0.648	0.676
	LA	0.259	0.255	0.259	0.258	0.233	0.246	0.223	0.256	0.250	0.259	0.195	0.262
	LL	0.272	0.269	0.269	0.271	0.247	0.252	0.256	0.261	0.268	0.257	0.268	0.275
	LW	0.322	0.409	0.406	0.411	0.326	0.362	0.369	0.373	0.347	0.405	0.395	0.416
	PL	0.361	0.393	0.397	0.399	0.367	0.371	0.405	0.394	0.394	0.371	0.364	0.407
	PN	0.282	0.294	0.298	0.302	0.257	0.276	0.283	0.287	0.289	0.280	0.258	0.303
	PH	0.740	0.769	0.756	0.759	0.748	0.744	0.763	0.764	0.762	0.769	0.755	0.777
Rice for Hangzhou	CL	0.777	0.788	0.773	0.782	0.782	0.772	0.779	0.783	0.784	0.770	0.786	0.792
	GY	0.304	0.306	0.309	0.307	0.307	0.296	0.300	0.296	0.307	0.293	0.307	0.315

	HD	0.571	0.534	0.497	0.498	0.498	0.524	0.514	0.525	0.536	0.543	0.548	0.550
	LA	0.197	0.185	0.195	0.195	0.195	0.140	0.179	0.170	0.189	0.199	0.130	0.201
	LL	0.262	0.268	0.271	0.275	0.275	0.264	0.255	0.244	0.269	0.272	0.264	0.277
	LW	0.272	0.295	0.314	0.305	0.305	0.267	0.242	0.287	0.276	0.267	0.285	0.307
	PL	0.286	0.314	0.321	0.321	0.321	0.297	0.278	0.304	0.305	0.318	0.302	0.322
	PN	0.177	0.186	0.182	0.183	0.183	0.174	0.172	0.169	0.189	0.168	0.174	0.196
	PH	0.771	0.776	0.768	0.774	0.774	0.768	0.763	0.771	0.776	0.759	0.773	0.782
	DTT	0.827	0.872	0.882	0.895	0.883	0.880	0.875	0.888	0.894	0.891	0.895	0.898
Maize	EW	0.547	0.589	0.592	0.613	0.564	0.604	0.579	0.603	0.602	0.606	0.600	0.617
	PH	0.849	0.880	0.876	0.891	0.883	0.880	0.884	0.887	0.888	0.883	0.869	0.895
	GY1	0.280	0.281	0.168	0.251	0.200	0.276	0.249	0.216	0.274	0.277	0.273	0.282
Wheat	GY2	0.184	0.160	0.165	0.217	0.188	0.240	0.203	0.219	0.224	0.252	0.264	0.294
	GY3	0.113	0.125	0.061	0.126	0.072	0.134	0.113	0.126	0.140	0.105	0.132	0.146
	GY4	0.102	0.180	0.075	0.195	0.158	0.175	0.146	0.164	0.172	0.178	0.182	0.197

Supplementary Table 2| Comparative evaluation of prediction errors with three representative phenotypes for MeNet, rrBLUP, and DeepCCR.

Phenotype	Model	NMSE	MAPE	RRMSE
GY	MeNet	0.643	0.270	0.394
	rrBLUP	0.686	0.272	0.396
	DeepCCR	0.660	0.280	0.406
GPC	MeNet	0.687	0.049	0.069
	rrBLUP	0.697	0.050	0.070
	DeepCCR	0.698	0.050	0.070
HD	MeNet	0.275	0.040	0.060
	rrBLUP	0.349	0.046	0.067
	DeepCCR	0.290	0.047	0.061

Supplementary Table 3| Number of remaining SNPs under different LD filtering thresholds.

r^2 threshold	0.1	0.2	0.3	0.4	0.5
Retained SNP count	2280	5885	14385	25556	43660

Supplementary Table 4| The mean wall times (s) for trait prediction with seven deep neural networks.

Model	GY	HD	LA	Mean
MeNet	11.304	10.066	9.037	10.136
VMGP	7.155	6.988	5.973	6.705
DeepCCR	8.321	7.280	7.101	7.567
SoyDNGP	22.444	24.789	22.832	23.355
WheatGP	9.399	8.943	8.848	9.063
GPformer	23.755	23.717	23.316	23.596
Cropformer	7.346	7.188	6.244	6.926

Supplementary Table 5| Ablation experiments on 12 rice traits from the Shanghai validate the effectiveness of VE and RepGeno.

Phenotype	VE	RepGeno	Phenotype	VE	RepGeno
CL	0.828	0.805	LL	0.351	0.347
GL	0.703	0.661	LW	0.445	0.166
GW	0.693	0.612	PL	0.416	0.400
GY	0.345	0.328	PN	0.287	0.272
HD	0.719	0.659	PH	0.809	0.794
LA	0.181	0.137	GPC	0.306	0.229

Supplementary Table 6| Reference hyperparameter configurations for MeNet on the phenotypic data of 12 rice traits from Shanghai.

Phenotype	bath size	learning rate	channels of VE	embedding dim of VE	embedding dim of RepGeno	margin
CL	32	0.01	[1, 4, 16]	[1024, 1024]	[8192, 1024]	0.4
GL	32	0.01	[1, 4, 16]	[4096, 1024]	[4096, 1024]	0.1
GW	128	0.01	[1, 1, 1]	[8192, 1024]	[8192, 1024]	0.1
GY	128	0.01	[1, 1, 1]	[8192, 1024]	[8192, 1024]	0.1
HD	128	0.01	[8, 16, 64]	[1024, 1024]	[8192, 1024]	0.3
LA	128	0.01	[1, 1, 1]	[8192, 1024]	[8192, 1024]	0.1
LL	128	0.01	[2, 4, 8]	[1024, 1024]	[8192, 1024]	0.2
LW	128	0.01	[1, 1, 1]	[8192, 1024]	[8192, 1024]	0.1
PL	128	0.01	[2, 4, 8]	[1024, 1024]	[8192, 1024]	0.2
PN	128	0.01	[2, 4, 8]	[1024, 1024]	[8192, 1024]	0.1
PH	32	0.01	[1, 4, 16]	[1024, 1024]	[8192, 1024]	0.1
GPC	128	0.01	[1, 1, 1]	[8192, 1024]	[8192, 1024]	0.1

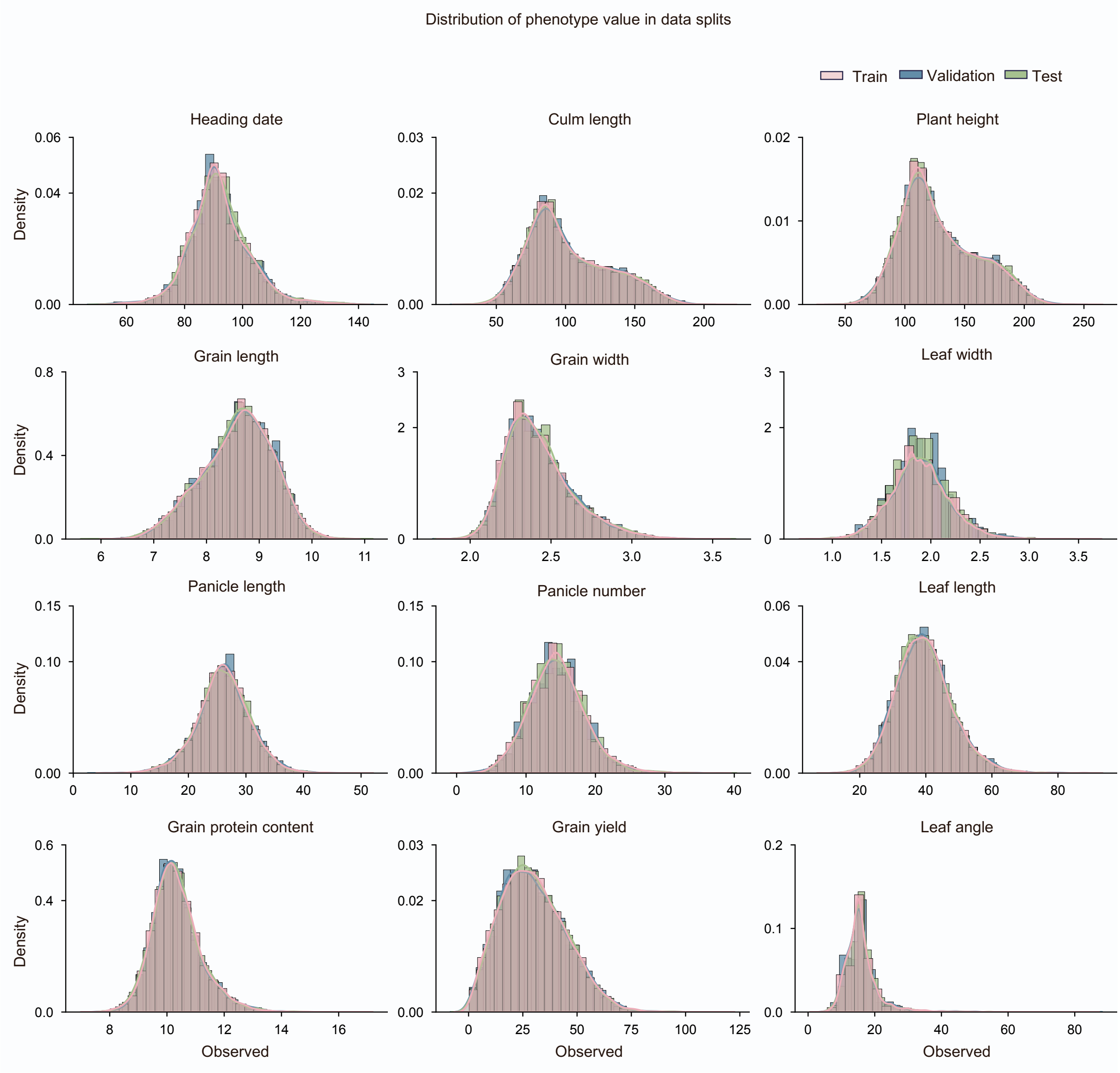

Supplementary Figure 1. Distribution comparison of each phenotype across training, validation, and test sets in the Shanghai dataset. Histograms represent frequency distribution, while KDE curves provide smoothed probability density estimates.

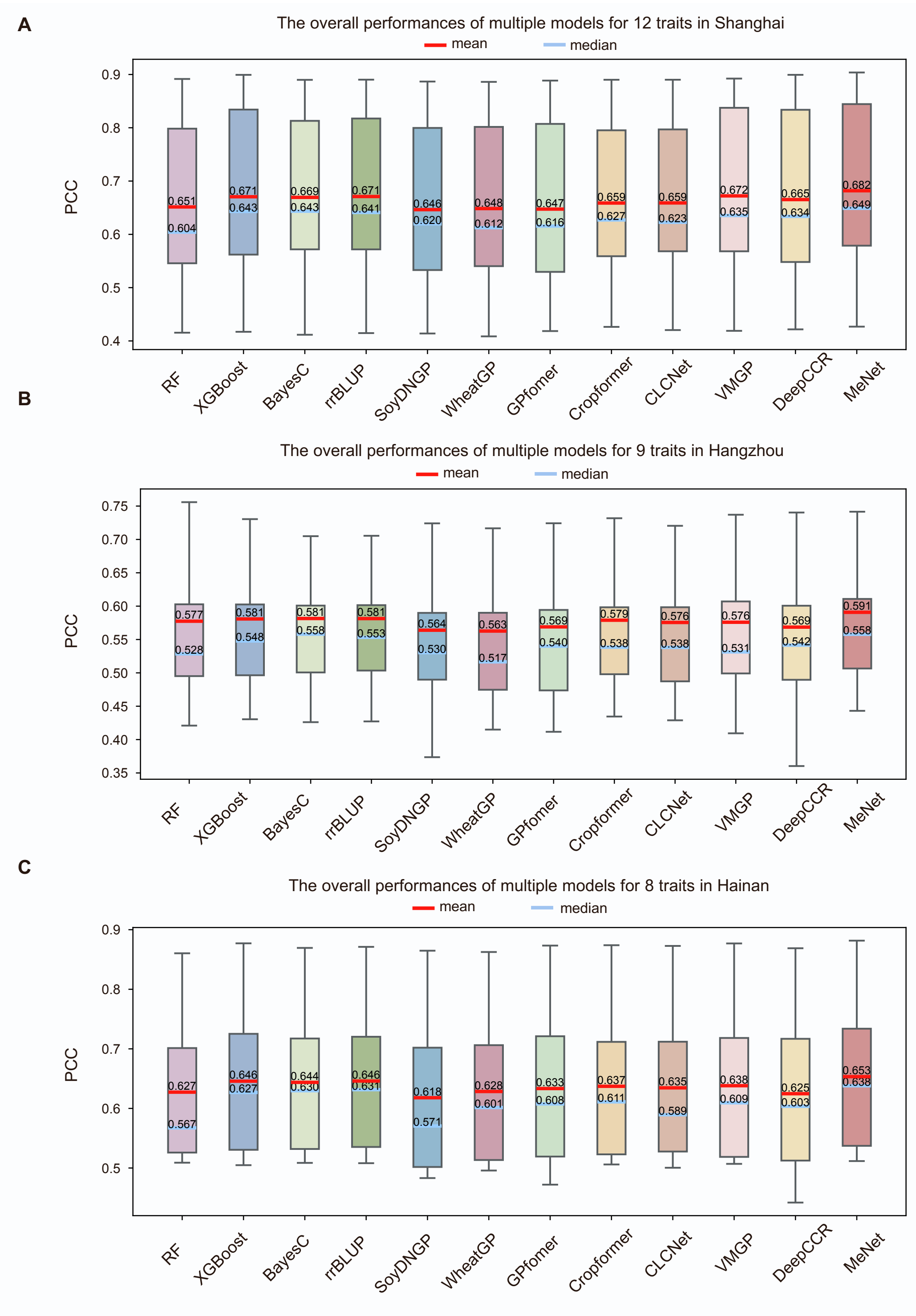

Supplementary Figure 2. Boxplots of the Pearson correlation coefficient (PCC) across multiple models for 12 traits in Shanghai (A), 9 traits in Hangzhou (B), and 8 traits in Hainan (C), using a 6:2:2 split for train set: validation set: test set. The red and blue lines indicate the mean and median PCC values, respectively.

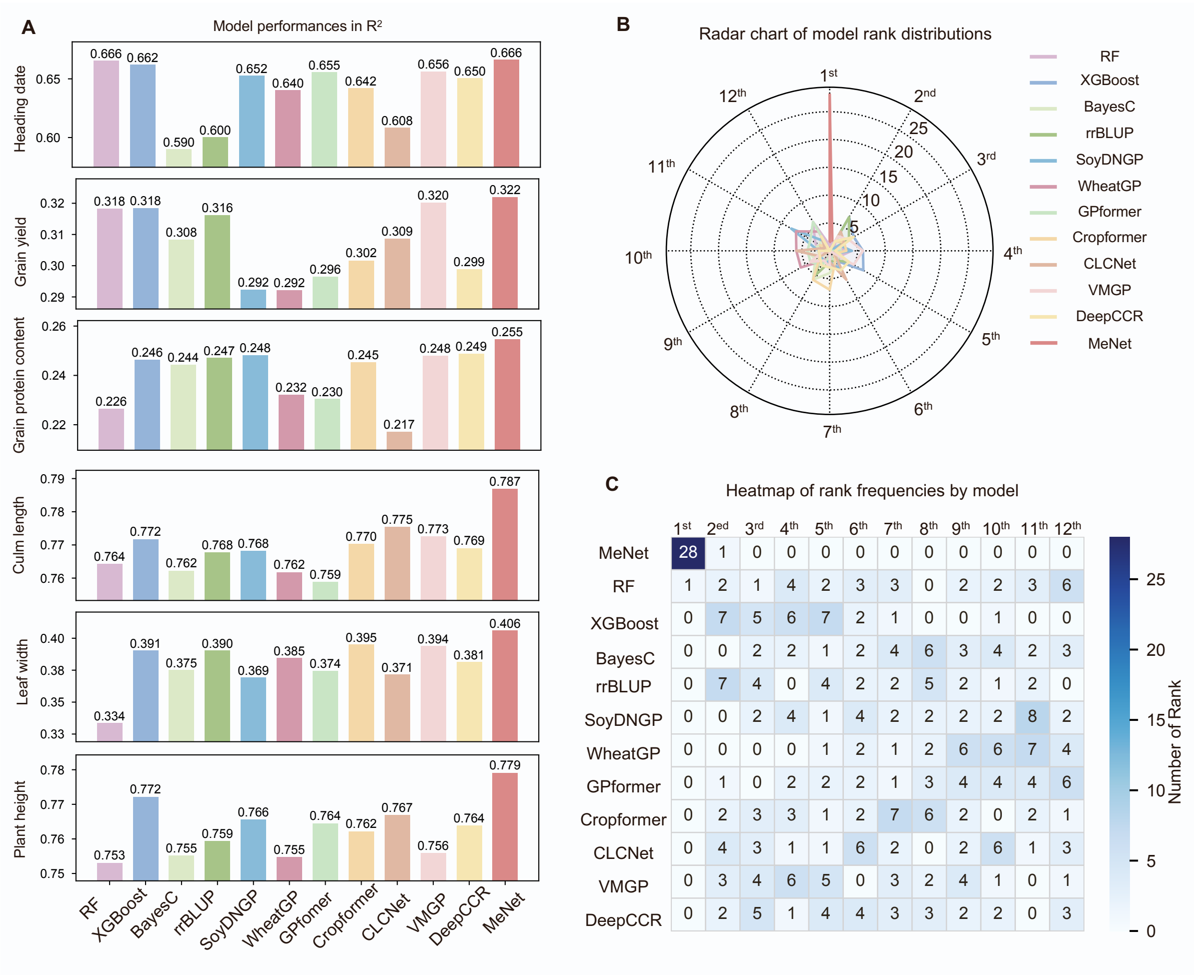

Supplementary Figure 3. Performances of MeNet for multiple rice traits under 3 environments with 3:2:5 for train set: validation set: test set. A, Bar charts showing the predictive ability (R^2) of MeNet compared with RF, XGBoost, BayesC, rrBLUP, SoyDNGP, WheatGP, GPformer, Cropformer, CLCNet, VMGP, and DeepCCR across rice traits in Shanghai. B-C, Statistical plots of ranks of predictive performances in R^2 for 29 tests by 11 models in the radar chart (B) and the heatmap (C).

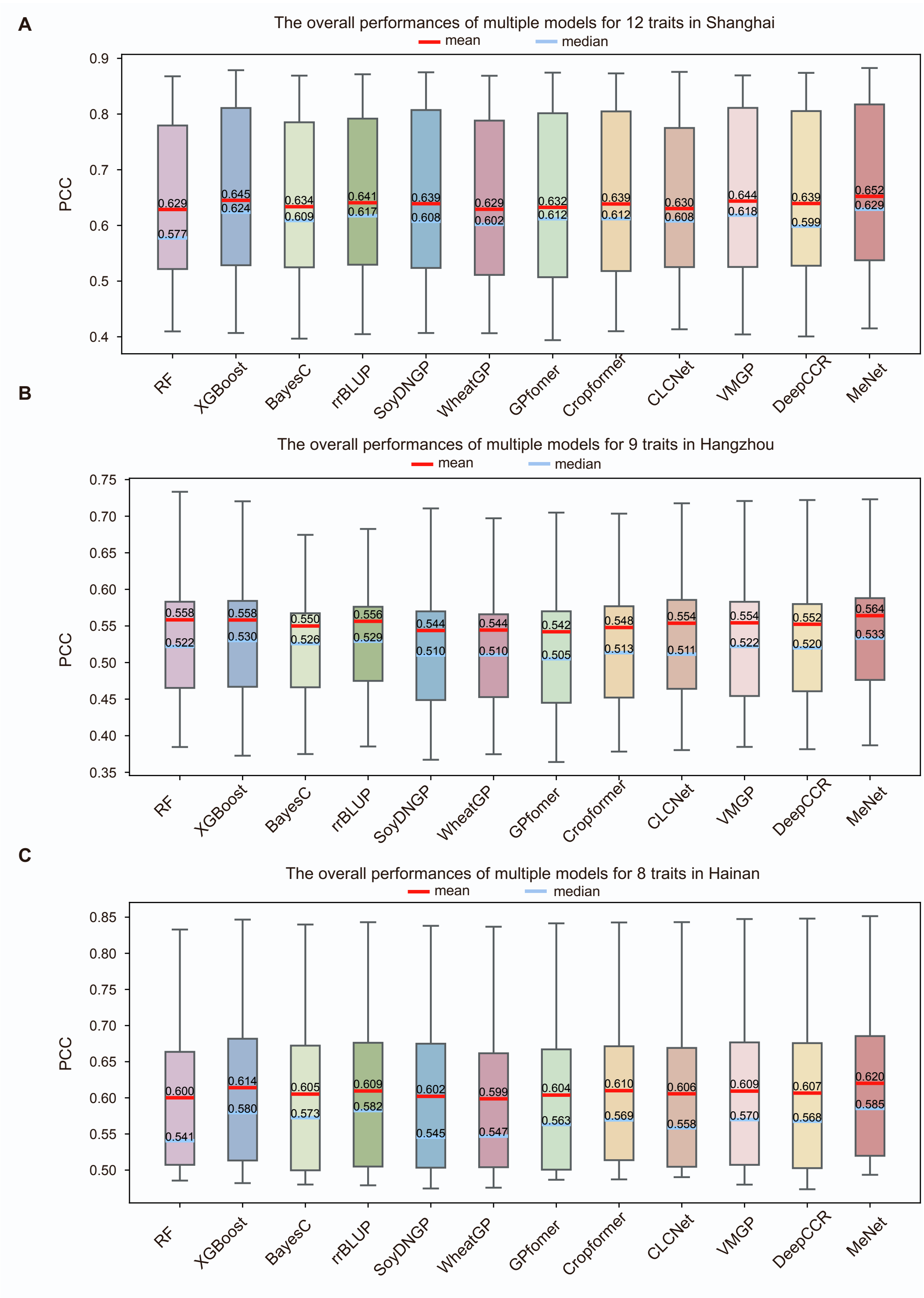

Supplementary Figure 4. Boxplots of the Pearson correlation coefficient (PCC) across multiple models for 12 traits in Shanghai (A), 9 traits in Hangzhou (B), and 8 traits in Hainan (C), using a 3:2:5 split for train set: validation set: test set. The red and blue lines indicate the mean and median PCC values, respectively.

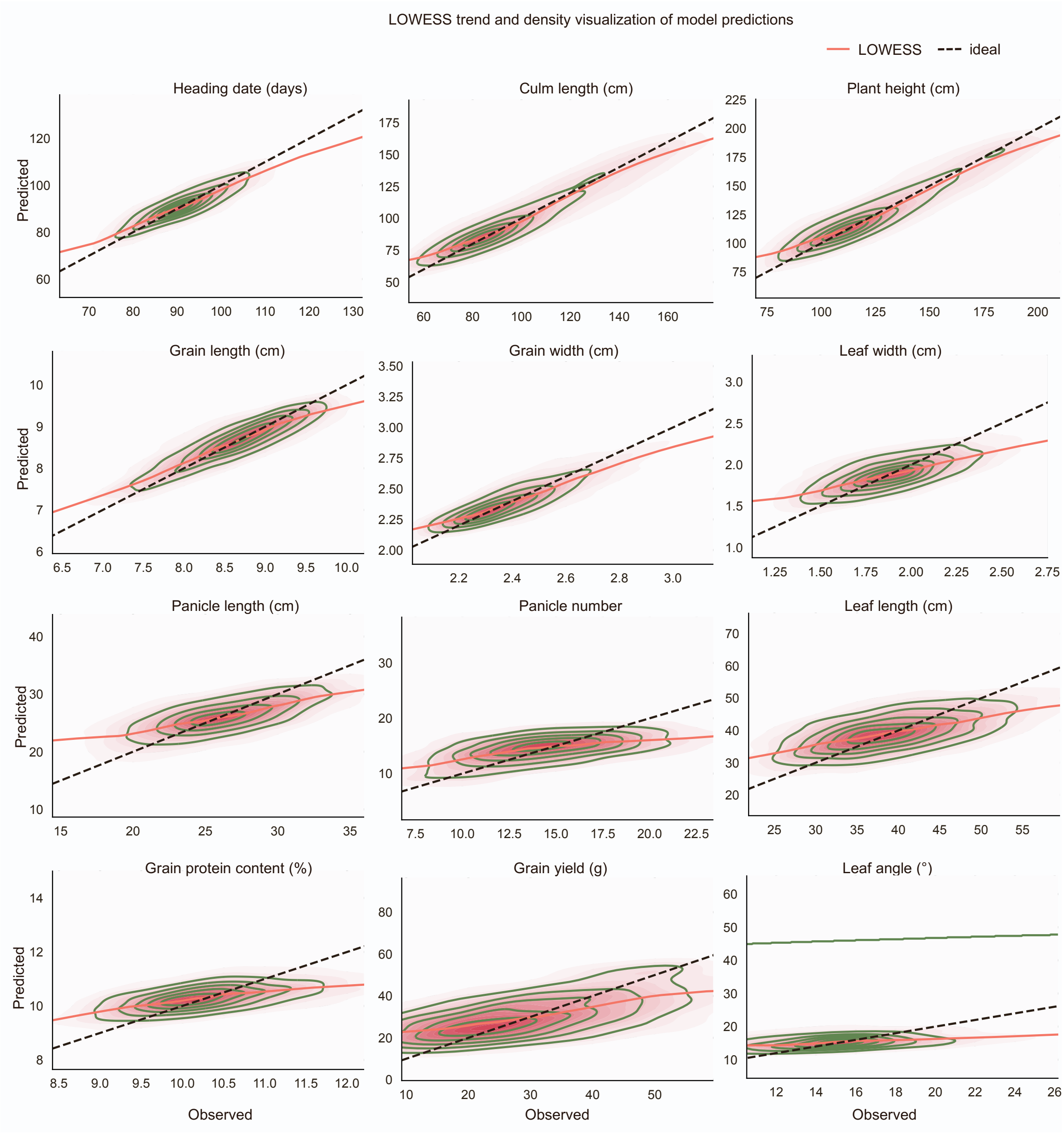

Supplementary Figure 5. The background is a 2D kernel density heatmap indicating data distribution in Shanghai dataset. The red curve represents the LOWESS fit, and the black dashed line indicates the ideal prediction ($y = x$).

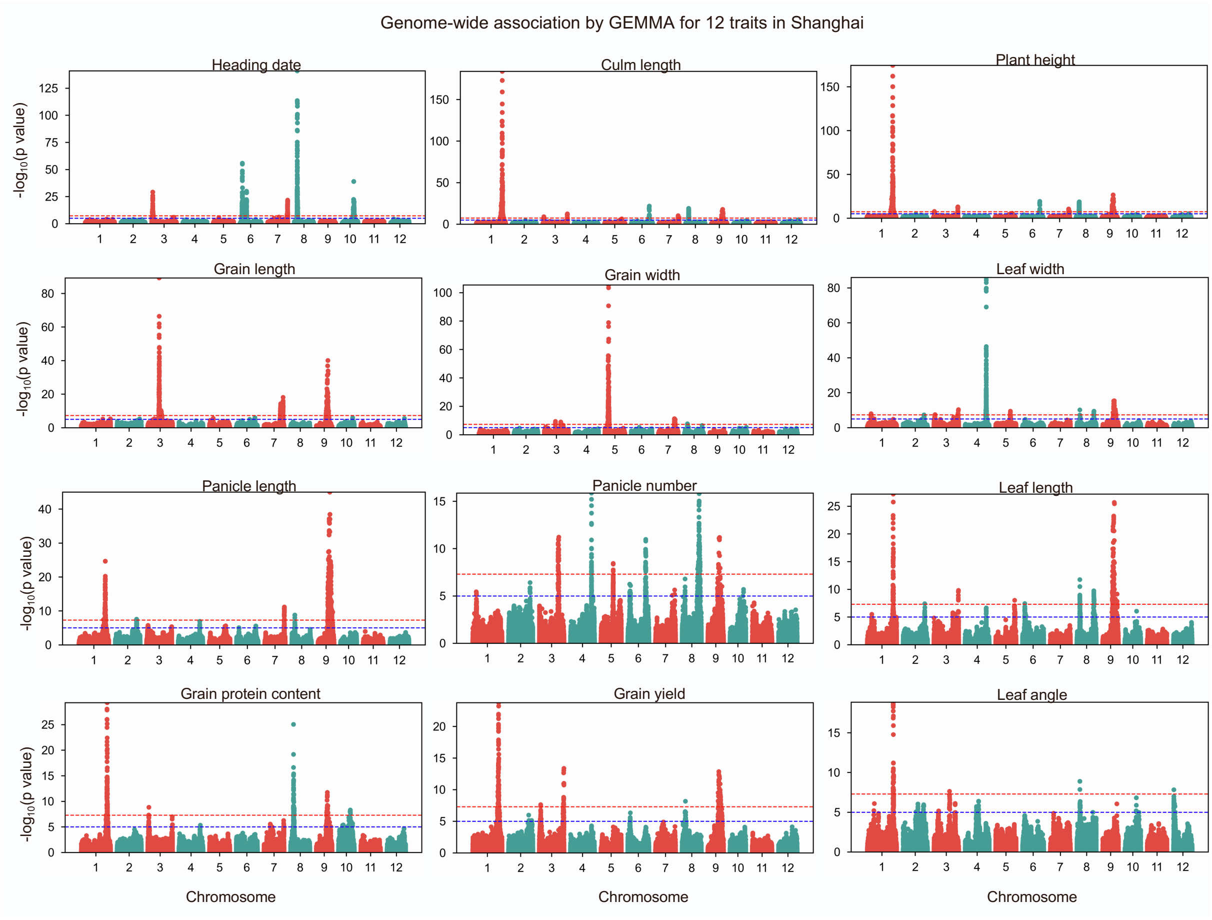

Supplementary Figure 6. Manhattan plots of genome-wide association analyses performed by GEMMA for 12 traits in Shanghai.

MeNet Software tools for Agronomic Trait Prediction

The MeNet software integrates model training, prediction, and transfer learning within a unified framework. It supports custom data import, hyperparameter configuration, and model saving/loading. With an intuitive graphical user interface, the software streamlines the end-to-end modeling and inference workflow, eliminating the need for programming skills. Tailored for crop breeding applications, it significantly enhances selection efficiency and operational simplicity.

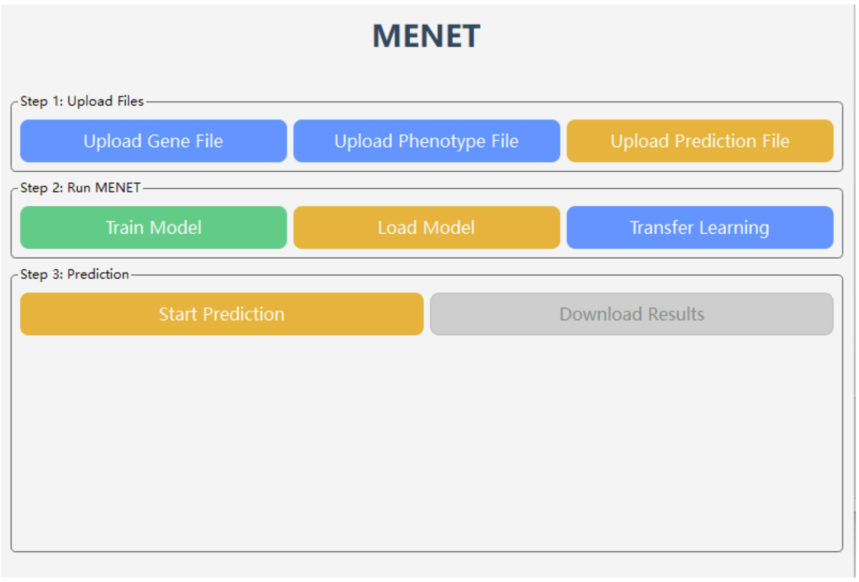

Supplementary Figure 7. The MeNet software user interface provides an intuitive and visually streamlined platform that facilitates seamless model training, dataset import, hyperparameter tuning, and inference.

Model training and saving

The software features a visual interface that enables users to flexibly configure training-related hyperparameters and import custom datasets. After training, the resulting models can be saved for subsequent loading, deployment, or transfer learning purposes.

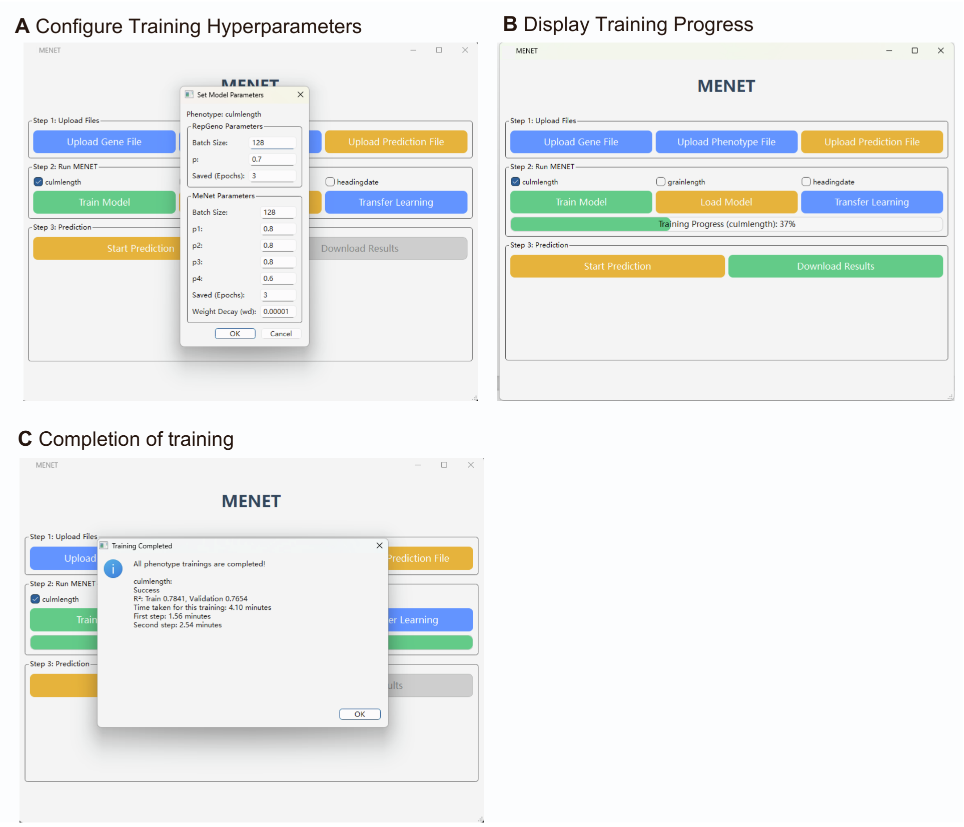

Supplementary Figure 8. (A) Hyperparameter configuration interface. (B) Visualization of training progress. (C) Notification indicating completion of training.

Model loading and inference

The software supports loading previously trained model files, enabling users to rapidly perform inference. Once a model is loaded, forward inference is automatically executed, and the prediction results are promptly displayed.

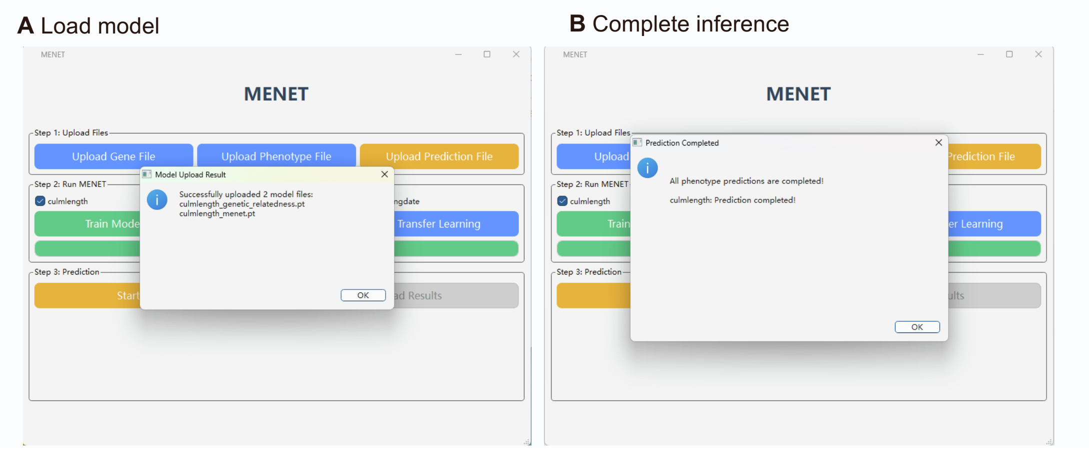

Supplementary Figure 9. (A) Interface for loading trained models. (B) Automatic inference.

Transfer Learning

The software enables transfer learning by allowing users to load previously trained models and fine-tune them on new datasets. Through a visual interface, users can configure parameters specific to the transfer learning process, which can be initiated with minimal effort. Upon completion, the updated model is automatically saved for future use and deployment.

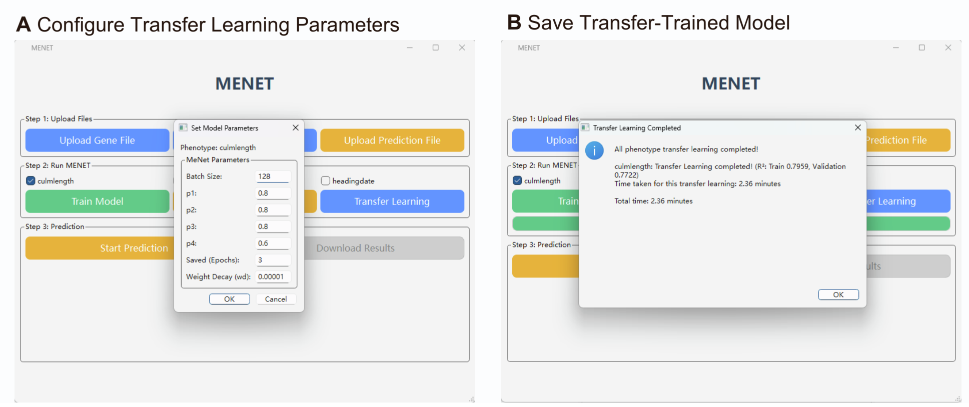

Supplementary Figure 10. (A) Interface for configuring transfer learning parameters. (B) Model saving after transfer learning.
